# Assembly and operation of an imaging system for long-term monitoring of bioluminescent and fluorescent reporters in plants

**DOI:** 10.1186/s13007-023-00997-0

**Published:** 2023-03-01

**Authors:** Maria L. Sorkin, Kathleen K Markham, Stevan Zorich, Ananda Menon, Kristen N. Edgeworth, Angela Ricono, Douglas Bryant, Rebecca Bart, Dmitri A. Nusinow, Kathleen Greenham

**Affiliations:** 1 Donald Danforth Plant Science Center, St. Louis, MO USA; 2 Department of Biological and Biomedical Sciences, Washington University in St. Louis, St. Louis, MO USA; 3 University of Minnesota, St. Paul, MN USA; 4 NewLeaf Symbiotics, St Louis, MO USA

**Keywords:** Bioluminescence, Fluorescence, Imaging, Non-invasive, Reporter assay, Luciferase

## Abstract

**Background:**

Non-invasive reporter systems are powerful tools to query physiological and transcriptional responses in organisms. For example, fluorescent and bioluminescent reporters have revolutionized cellular and organismal assays and have been used to study plant responses to abiotic and biotic stressors. Integrated, cooled charge-coupled device (CCD) camera systems have been developed to image bioluminescent and fluorescent signals in a variety of organisms; however, these integrated long-term imaging systems are expensive.

**Results:**

We have developed self-assembled systems for both growing and monitoring plant fluorescence and bioluminescence for long-term experiments under controlled environmental conditions. This system combines environmental growth chambers with high-sensitivity CCD cameras, multi-wavelength LEDs, open-source software, and several options for coordinating lights with imaging. This easy-to-assemble system can be used for short and long-term imaging of bioluminescent reporters, acute light-response, circadian rhythms, delayed fluorescence, and fluorescent-protein-based assays in vivo.

**Conclusions:**

We have developed two self-assembled imaging systems that will be useful to researchers interested in continuously monitoring in vivo reporter systems in various plant species.

**Supplementary Information:**

The online version contains supplementary material available at 10.1186/s13007-023-00997-0.

## Background

Long-term imaging allows for the real-time study of complex spatial and temporal biological phenomena in plants such as growth, stress responses, disease progression, and circadian rhythms [1–3]. Using fluorescent or bioluminescent reporters facilitates non-invasive monitoring and measurement of physiology and genetic screens in plants. Bioluminescent reporters, such as the firefly luciferase (*LUC*) gene, have been used to characterize circadian clock mutants [4, 5] or measure plant root growth in soil systems [6]. The wide use of *LUC* is due to the short half-life of the *LUC* mRNA and protein when the substrate luciferin is present [4]. The catalysis of luciferin and oxygen to oxyluciferin and CO_2_ is an ATP-dependent reaction that produces light emission at 560 nm [7]. A charge-coupled device (CCD) camera can detect this light emission and monitor the activity of any gene promoter or protein fused to the *LUC* gene in real time. The repeated measurement required for longitudinal studies is aided by systems that combine growth chambers and imaging equipment for autonomous data collection over long periods. While integrated commercial systems exist, they are often costly and may not have the flexibility that custom systems can provide.

We have developed two custom, open-source imaging, and illumination systems to non-invasively monitor plant bioluminescence and fluorescence. These systems combine environmental chambers, LED lighting, a CCD camera, a computer, and open-source software. We provide several options for customizing lighting and image acquisition to monitor natural and transgene-based bioluminescent and fluorescent signals in plants for both single time-point and long-term (≥ 2 weeks) analysis. This report describes how to assemble, program, and image bioluminescence and fluorescence to study acute responses to the environment, circadian rhythms, and plant-microbe interactions.

## Results

### Imaging system set-up

#### Ultrasensitive CCD camera system

Imaging bioluminescence from transgenic organisms requires a lengthy exposure to detect the dim signal. Ultrasensitive, cooled CCD camera systems can maximize the observable range of reporter strength. To monitor luciferase reporter activity, we use the high quantum efficiency and ultralow-noise 1024b Pixis CCD camera (Princeton Instruments). Others have set up similar systems with an Andor iKon-M 934 (Oxford Instruments, see Methods). We use a Navitar NMV-25M1 (25.0 mm Focal length, f/1.4) lens mounted on the camera to manually alter the focal length depending on the field of view. If fluorescence imaging is also desired, consider lenses compatible with screw-on filters.

#### LED lighting for plant growth and imaging

Long-term imaging of plants requires a suitable light source to support plant growth. The ability to illuminate plants with specific light wavelengths is essential to study light signaling pathways. Both fluorescent and white light-emitting diodes (LEDs) can be used to grow plants, and an array of filters can modify the light source's spectral qualities. However, the phosphor compounds in fluorescent and white-light LEDs that produce their broad spectrum emit light long after they are off, thereby reducing the imaging sensitivity. To avoid extending the dark period to minimize background from phosphorescence, single-wavelength LED arrays are an alternative for plant growth that provides hue control. Single-wavelength LEDs can also be used as sources for fluorescent imaging when combined with appropriate lens filters. Furthermore, single-wavelength LEDs can be rapidly turned on and off without subsequent phosphorescence, allowing for millisecond timing between illumination and imaging. Therefore, when assembling a lighting system for a long-term autonomous illumination and imaging system, we chose multiple single-wavelength LED arrays for lighting.

**Fig. 1:**
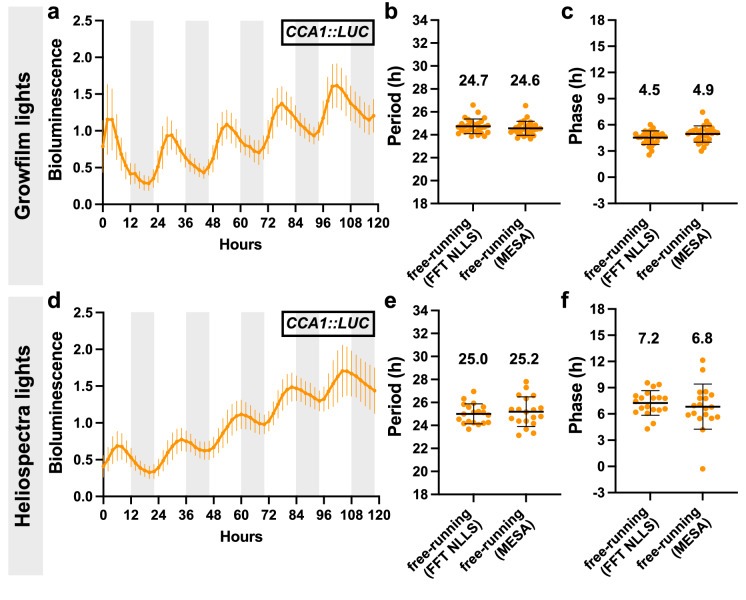
Arabidopsis seedlings under free-running conditions in white or single-wavelength LED lights. **a**–**c** Analysis of bioluminescent traces from *CCA1pro::LUC* seedlings grown, entrained, and imaged using white LED (Growfilm) lights. Ten day old seedlings were grown and entrained under a 12 h:12 h light:dark and 20 °C:16 °C hot:cold (step) cycle and then released into free-running conditions (constant light with PPFD of ~ 150 µmol m^−2^ s^−1^, constant 20 °C, constant ~ 40% relative humidity) for 5 days. **a** Average of *CCA1pro::LUC* traces from 28 seedlings under free-running conditions (error bars represent SD). Light gray boxes represent subjective night hours. Each seedling was normalized to its own average bioluminescence over time. **b**–**c** Method-specific period (**b**) and method-specific circadian phase (**c**) of 28 seedlings from (**a**), as reported by BioDare2 using FFT-NLLS or MESA analysis methods (error bars represent SD). Average period and phase from 28 seedlings are reported above data points. **d**–**f** Analysis of bioluminescent traces from *CCA1pro::LUC* seedlings grown, entrained, and imaged using single-wavelength (Heliospectra) lights. Ten day old seedlings were grown and entrained under a 12 h:12 h light:dark and 20 °C:16 °C hot:cold (step) cycle and then released into free-running conditions (constant 22 °C, constant light with PPFD of ~ 120 µmol m^−2^ s.^−1^, constant ~ 25% relative humidity) for 5 days. **d** Average of *CCA1pro::LUC* traces from 19 seedlings under free-running conditions (error bars represent SD). Each seedling was normalized to its own average bioluminescence over time. **e**–**f** Method-specific period (**e**) and method-specific circadian phase (**f**) of 19 seedlings from (**d**) (error bars represent SD)

We recognize that single-wavelength LED arrays can be expensive and white LEDs might be the only option for some users. If white LEDs are used, an extended dark period is required before imaging to allow the phosphorescence to dissipate. To compare the imaging conditions between white LEDs and single-wavelength Heliospectra lights, we imaged Arabidopsis seedlings expressing the *CIRCADIAN CLOCK ASSOCIATED 1* promoter fused to *LUCIFERASE* (*CCA1pro::LUC)* alongside wild-type Columbia (Col) seedlings with increasing dark periods before imaging. A dark period of 10 min was needed before the acquisition of the image to eliminate the phosphorescence effect from the white LEDs. We captured robust rhythms of *CCA1pro::LUC* that gave the expected period of 24.5 h and a phase of ZT5, demonstrating that the use of white LEDs is acceptable as long as phosphorescence is diminished (Fig. 1). During both imaging experiments we monitored the actual temperature in the chamber using a data logger and found that the Heliospectra lights did produce slightly more heat than the white LEDs (Additional file 3: Figure S1). However, the chamber was able to compensate for this and maintain a temperature within 2 °C of the set point. We recommend including an additional temperature sensor within the chamber to have a more accurate record of the actual temperature throughout the experiment, and to adjust the setpoint of the chamber to accommodate for the additional heat load.

Of the available systems, Heliospectra lights have key attributes that facilitate integration into an imaging system. These lights provide multiple wavelength configurations and monitoring over a Web User Interface, and the user can control the daily timing of the hue and intensity of the light source. Details for programming and controlling the timing of the lights are included in the Methods.

#### Climate-controlled imaging chamber

Using ultrasensitive camera systems to image plants requires the minimization of all extraneous light sources. Many available environmental chambers can be modified into a controlled-environment imaging system. To test the level of light pollution within a chamber, place the CCD camera inside and begin a long exposure at the most sensitive setting. The resulting image can be compared to an equivalent exposure with the lens cap on to compare as a dark baseline. Any difference between the images indicates an unwanted source of light. If light is leaking through chamber doors, weather stripping can be used to improve seals. Alternatively, chambers can be purchased with a secondary door as a modification that minimizes the light from outside. Unwanted light can also come from within the chamber if they are not made of unpainted stainless steel, as the painted interiors of many chambers contain phosphorescent compounds, which will emit light long after a light source has been turned off. Paint can also produce unwanted fluorescent signals during the imaging of biological fluorescence. Matte black paint (e.g. Rust-Oleum ChalkBoard paint) can eliminate unwanted phosphorescence/fluorescence signals from a painted chamber that would otherwise interfere with imaging. Painting the external surfaces of the lighting system with matte black paint or covering them with aluminum foil may also be required. Any exterior status lights should be covered or disabled on the lighting or imaging equipment if necessary.

**Fig. 2:**
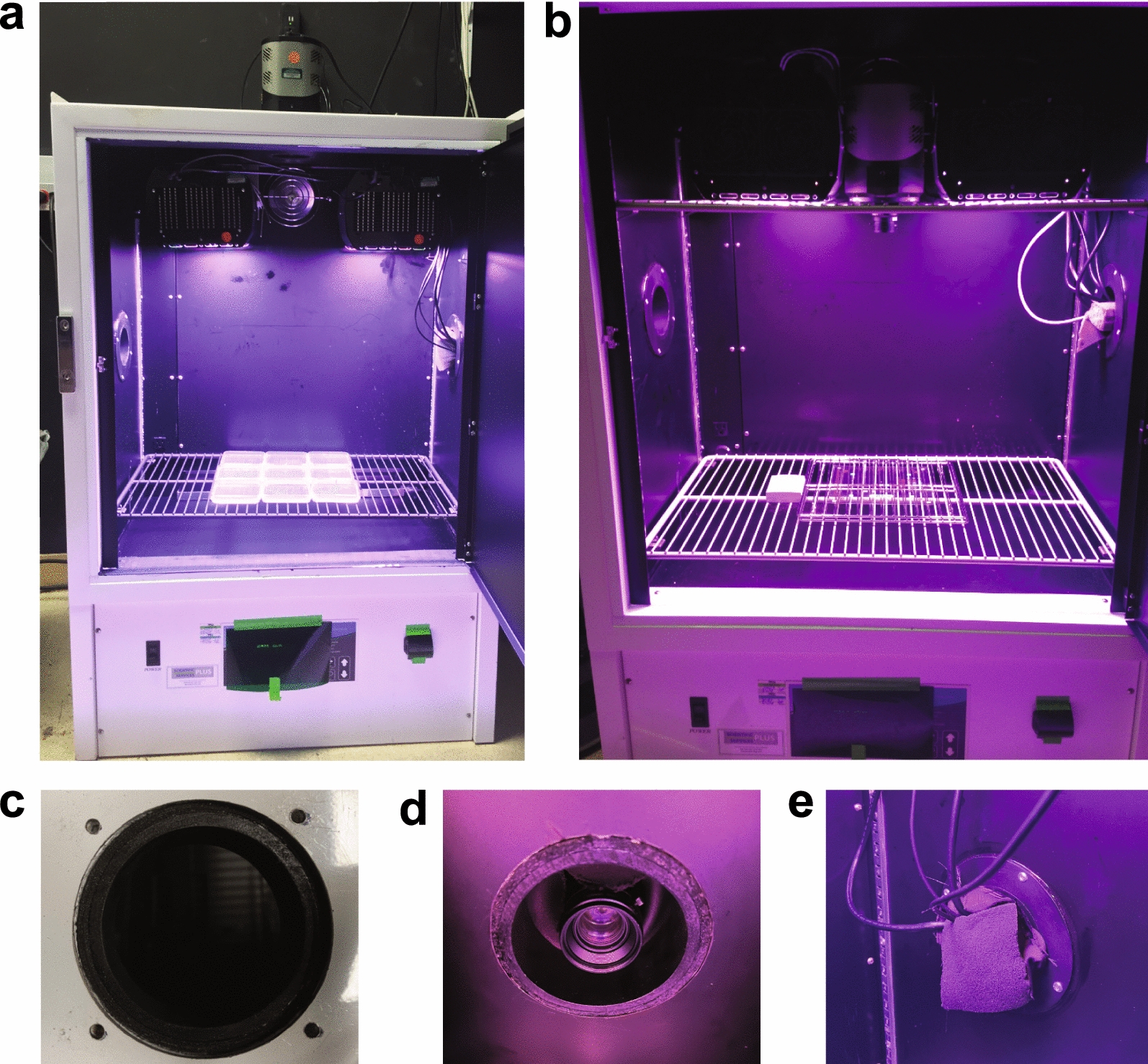
System 1 Chamber and light setup. **a** Chamber setup with camera mounted on top of the growth chamber from the outside. **b** Chamber setup with camera mounted on a wire rack inside of the growth chamber. **c** Close-up, top-down view of custom opening cut into top of growth chamber that holds camera in position. **d** Close-up, bottom-up view of camera when settled into custom opening shown in (**c**). **e** Close-up view of growth chamber port hole with wires feeding to outside. All spaces of the port hole are filled with black foam blocks to prevent light leakage from the dark room into the imaging chamber

**Fig. 3:**
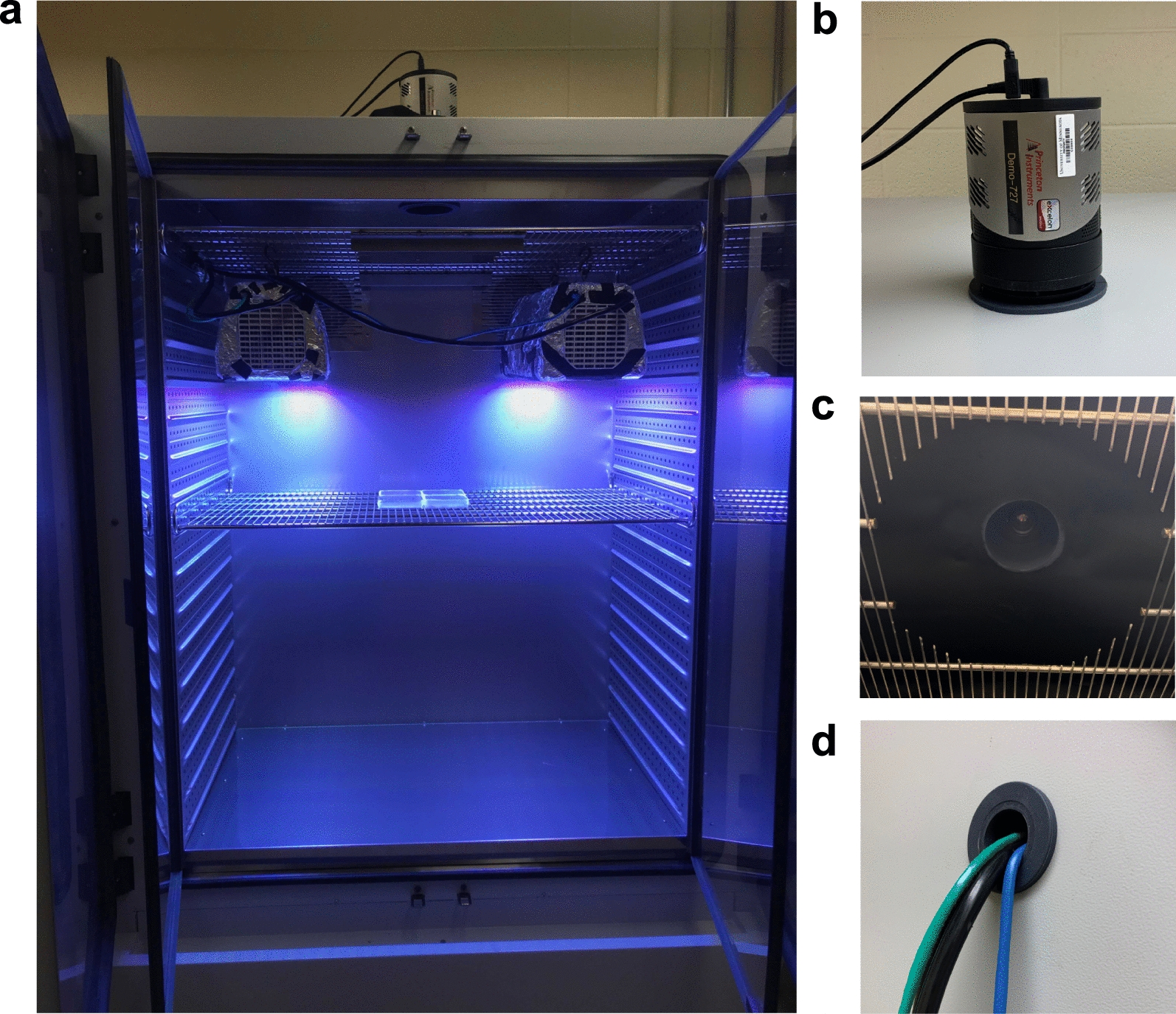
System 2 Chamber and light setup. **a** Binder chamber setup with camera mounted on top of chamber from the outside. **b** Close-up of the camera mounted on top of the chamber. **c** Close-up of the hole cut out of the shelf to provide a clear view for the camera lens. **d** Close-up view of chamber port hole with wires feeding to outside. All spaces of port hole are filled with black foam or foil to prevent light leakage from the dark room into the imaging chamber

The environmental chambers contain adjustable wire shelving that holds plant tissue culture plates or pots and can support the camera and lights (Figs. 2, 3). There are no specifications for the type of plates that can be used in either system. We do recommend black plates when possible to reduce any signal from neighboring plates or plants from being detected. The shelf for holding the camera and lights needs to be modified to allow the camera lens to pass through (see Methods) unless the spacing is sufficient. To mount a camera inside the chamber, use a bracket system for safety and to minimize movement and vibration. For example, we have used a 3D printed mount (painted or printed in black) to fasten the camera to the shelf (Fig. 2) (https://www.thingiverse.com/thing:432709). In later iterations and system build #2 (Fig. 3), we mounted the camera outside the chamber to prevent any humidity from within the chamber affecting the camera and to increase the field of view. We positioned the lights adjacent to the camera to minimize differences in illumination over the growing surface. Black foam and black cloth are used to prevent light leaking into the chamber from the side ports where the power and communication cords for the camera and lights enter (Fig. 3d). The samples are placed on the wire rack, which provides the necessary airflow to minimize condensation within tissue culture plates, or on the floor of the incubator. An alternative option for the imaging system is to mount the lights directly to the chamber's ceiling.

#### Acquiring images

All image acquisition and camera specifications were performed using the open-source µManager software (https://micro-manager.org/). The µManager software is used to set the time between acquisitions, which must be greater than the length of time of the exposure plus any delay before imaging. For bioluminescent imaging experiments, a three to five minute delay before imaging will reduce signal from delayed fluorescence (DF) from the plant photosystem [8]. Testing the system with non-bioluminescent plants can determine how long of a delay is required to minimize signals from DF. For non-photosynthetic organisms, this delay should be long enough to completely turn off the lights before opening the shutter of the camera–at least 10 ms. Plant auto-fluorescence is minimized by illuminating plant samples with 400 nm light to visualize Green Fluorescent Protein (GFP) signals. The excitation wavelength, fluorescent protein, filter, and the sample should all be optimized to maximize detection. The exposure time necessary to visualize a bioluminescent signal will vary depending on the reporter construct; a weak promoter driving *LUC* might require a ten minute exposure, while a strong promoter may only need three minutes.

### Time course imaging experiments

#### Bioluminescence imaging

Bioluminescent reporters allow for non-invasive monitoring of gene expression and have been used extensively in genetic screens [9–11]. We developed our imaging systems for long-term monitoring of *LUC* reporters driven by plant circadian clock-responsive promoters. We have analyzed the circadian phenotype of known and newly discovered genes with roles in regulating circadian rhythms [12]. A typical circadian imaging assay involves initial growth under a light/dark entrainment period. One day before imaging, luciferin is applied, and plants are transferred to the desired imaging conditions–typically constant light or darkness (Additional file 3: Figure S2). Images are taken every 1–2 h over 5–7 days to capture the reporter gene's rhythmic expression. The exposure time is determined based on the intensity of the reporter. For example, the *CCA1* promoter is highly active, requiring an exposure of ~ 1–3 min with the Pixis or Andor CCD camera systems (see Methods). In contrast, the *TIMING OF CAB EXPRESSION 1* (*TOC1*) promoter is weak and requires a ~ 3–5 min exposure time.

**Fig. 4:**
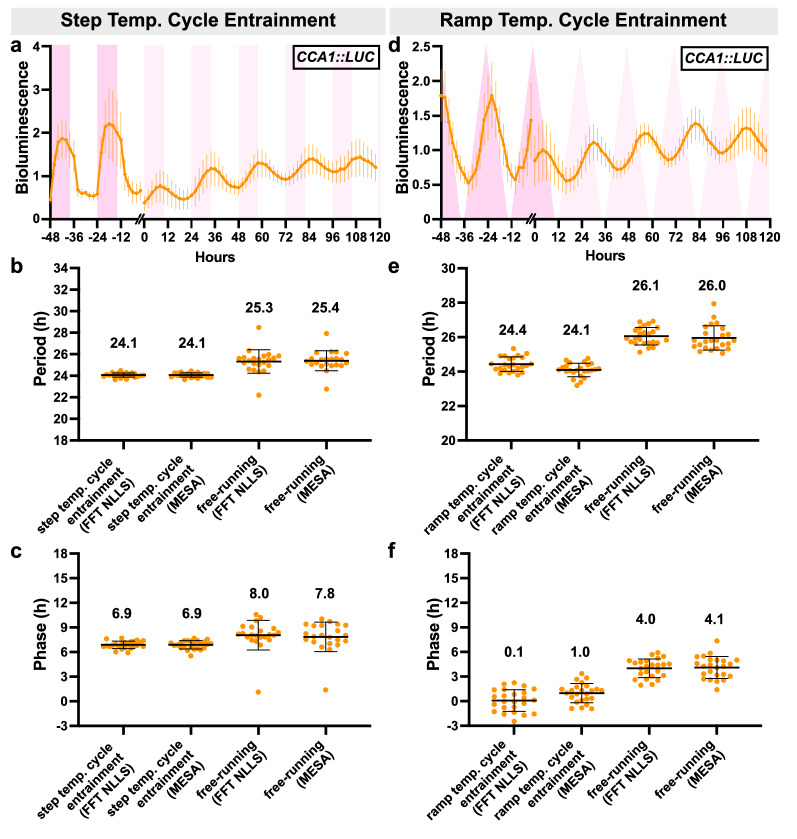
Arabidopsis *CCA1pro::LUC* expression following entrainment to step and ramp thermocycle conditions. **a** Bioluminescent traces of ten-day old *CCA1pro::LUC * seedlings (grown under a 12 h:12 h light:dark, fluorescent lights, 22 °C in a growth room) that were entrained in a step temperature cycle (22 °C:12 °C for 12 h:12 h, constant light with PPFD of ~ 120 µmol m^−2^ s^−1^, constant ~ 25% relative humidity) for 5 days and then released into free-running conditions (constant 22 °C, constant light with PPFD of ~ 120 µmol m^−2^ s^−1^, constant ~ 25% relative humidity) for 5 days. Dark red and light red boxes represent warm and subjective warm hours, respectively. Error bars represent SD from at least 23 seedlings. Each seedling was normalized to its own average bioluminescence over time. **b**–**c** Method-specific period (**b**) and method-specific circadian phase (**c**) of *CCA1pro::LUC* seedlings under step temperature cycle entrainment and free-running conditions, as reported by BioDare2 using FFT-NLLS or MESA analysis methods. Average period and phase are reported above data points. Error bars represent SD from at least 23 seedlings. For period analysis in BioDare2, “linear dtr” was selected for detrending, 18-34 h was selected for expected period, and “FFT NLLS” or “MESA” was selected for analysis method. **d** Bioluminescent traces of ten-day old *CCA1pro::LUC* seedlings (grown under a 12 h:12 h light:dark, fluorescent lights, 22 °C in a growth room) that were entrained in a ramp temperature cycle (22 °C:12 °C for 12 h:12 h, with 1-2 °C increments/h until reaching the set point, constant light with PPFD of ~ 120 µmol m^−2^ s^−1^, constant ~ 25% relative humidity) for 5 days and then released into free-running conditions (constant 22 °C, constant light with PPFD of ~ 120 µmol m^−2^ s^−1^, constant ~ 25% relative humidity) for 5 days. Error bars represent SD from 24 seedlings. Each seedling was normalized to its own average bioluminescence over time. **e**–**f** Method-specific period (**e**) and method-specific circadian phase (**f**) of *CCA1pro::LUC* seedlings under ramp temperature cycle entrainment and free-running conditions, as reported by BioDare2 using FFT-NLLS or MESA analysis methods. Error bars represent SD from 24 seedlings. For period analysis in BioDare2, “linear dtr” was selected for detrending, 18-34 h was selected for expected period, and “FFT NLLS” or “MESA” was selected for the analysis method

Using an environmental chamber for imaging allows us to examine reporter responses to temperature changes independently or alongside light conditions. To demonstrate the sensitivity to temperature, we imaged *CCA1pro::LUC* expressing Arabidopsis seedlings under thermocycle (12 h light 22 °C/12 h light 12 °C) entrainment followed by release into free-run (24 h light, constant 22 °C). We ran two thermocycle programs: a step- and a ramp- based temperature change. The step program changed immediately between the temperature set points (22 °C/12 °C) and the ramping program gradually increased the temperature by 1-2 °C every hour until reaching the set temperature (Fig. 4a, d). Rhythmic expression of the *CCA1* promoter was observed for both programs, with some variation in phase and period between the step and ramping protocol (Fig. 4).

**Fig. 5:**
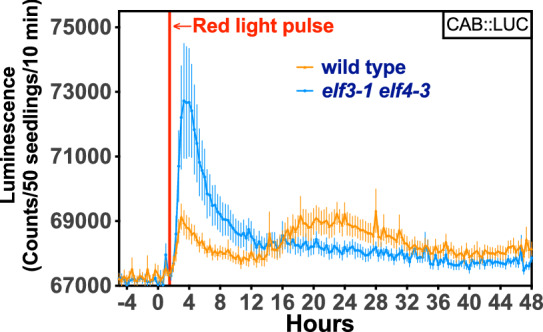
Acute light induction of luciferase reporter. A single red-light pulse (Heliospectra intensity value 200; 20 µmol m^−2^ sec.^−1^) was applied for 10 min to wild-type or *elf3-1 elf4-3* seedlings containing a *CABpro::LUC* transcriptional reporter construct. Error bars represent SD, n = 8

In addition to circadian assays, we can also measure acute light responses. By exposing dark-grown seedlings to a single red-light pulse, we observed a single acute peak of a *CHLOROPHYLL A/B BINDING (CAB)pro::LUC* reporter followed by a secondary circadian peak 24 h later in wild-type Arabidopsis (Fig. 5), which is lost in the arrhythmic circadian clock *elf3* (*EARLY FLOWERING 3)* mutant, as previously reported [13]. This flexible system can be programmed to autonomously monitor the response of bioluminescent reporters to a variety of light, temperature, and humidity conditions in the environmental chamber.

**Fig. 6:**
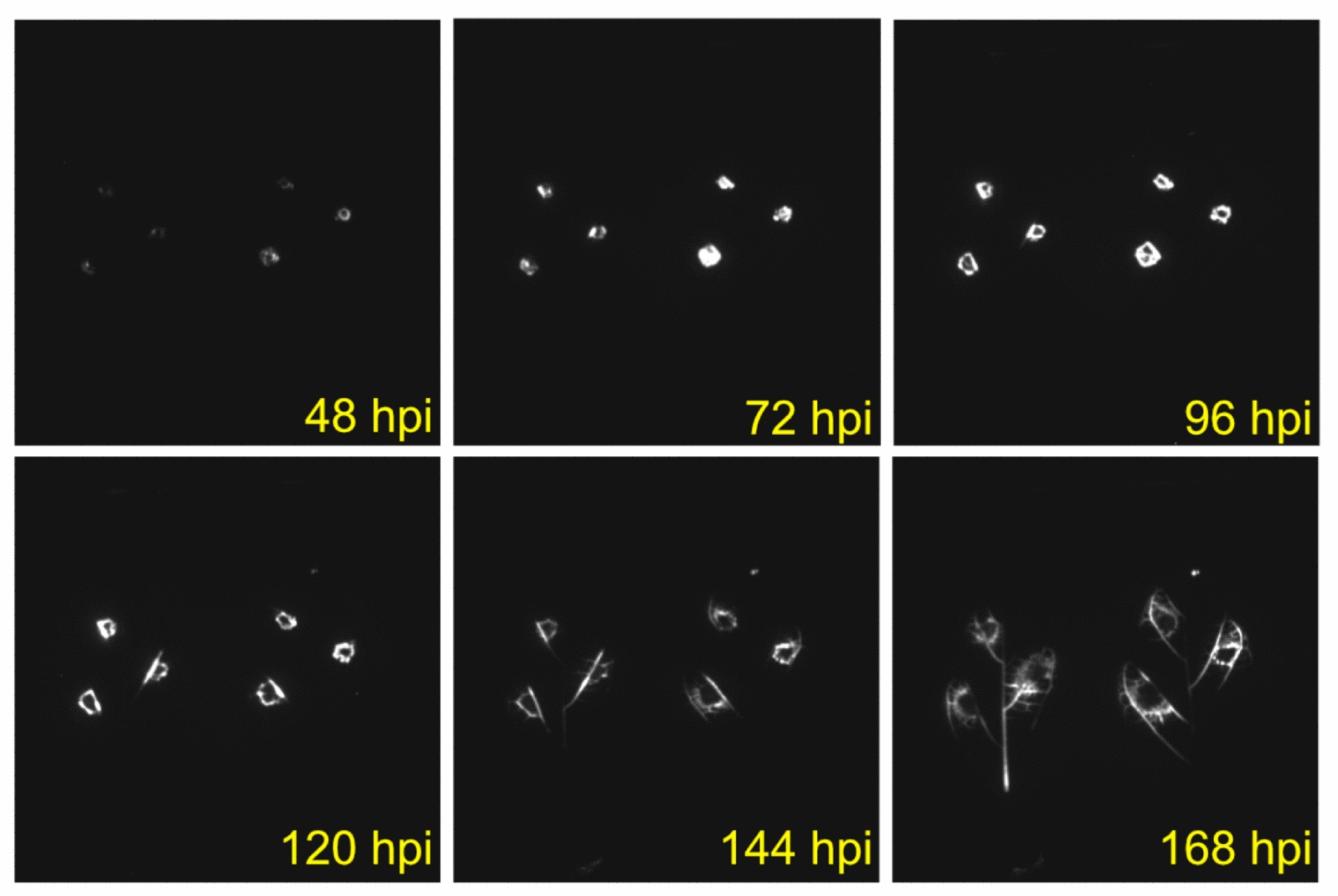
Xam infection on detached cassava leaves. *Xanthomonas phaseoli pv. manihotis* harboring lux^ABCDE^ plasmid was infiltrated into cassava leaves 48 h prior to detachment and imaged under light/dark cycles to 168 h post infiltration (hpi). Bacterial spread through vasculature is observed in later images

We also demonstrated the use of our system for studying plant–microbe interactions. As previously shown, the spread of a pathogen inside a plant host can be visualized using bacteria expressing a bioluminescent marker [2]. To test the ability of our custom imaging system to study pathogen infection, we infiltrated detached cassava leaves with *Xanthamonas phaseoli pv. manihotis* (previously called *X. axonopodis pv. manihotis*) expressing the *luxABCDE* transgene. We observed and measured the movement of this pathogen through detached leaves over time (Fig. 6). These methods can broadly dissect the role of the pathogen, host, and environment in the disease triangle. Alternatively, these methods can be used to monitor bioluminescent reporters in microorganisms in response to various environmental factors when grown independently from the host. For example, bacterial responses to light can be measured on petri dishes to determine how bacteria respond to environmental perturbations.

#### Delayed fluorescence

**Fig. 7:**
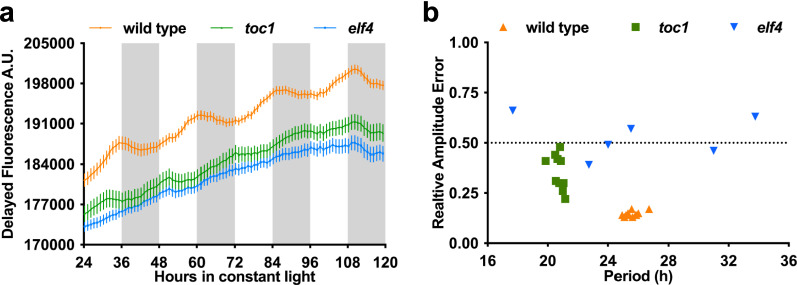
Delayed fluorescence of Arabidopsis seedlings. **a** Delayed fluorescence traces of six-day old wild-type, *toc1-4* (arrhythmic), and *elf4-2* (short period) seedlings grown in ~ 25 seedling patches (grown under a 12 h:12 h light:dark, fluorescent lights, 22 °C) released into free-running conditions (constant 22 °C, constant light with PPFD of ~ 70 µmol m^−2^ s^−1^, constant ~ 25% relative humidity) for 5 days. White and grey boxes represent subjective light and subjective night, respectively. Error bars represent SD from 12 patches of seedlings. Each patch was normalized to its own average bioluminescence over time. **b** Relative amplitude error (RAE) plot of measured rhythms from wild type, *elf4-2*, and *toc1-4* seedlings. RAE ≤ 0.5 is considered a robust waveform. 100% of wild type, 25% of *toc1-4,* and 8.33% of the *elf4-2* seedlings were below the 0.5 threshold. Seedling period under free-running conditions, as reported by BioDare2 using FFT-NLLS analysis methods. For period analysis in BioDare2, “linear dtr” was selected for detrending, 18–34 h was selected for expected period, and “FFT NLLS” was selected for analysis method. Each seedling was normalized to its own average bioluminescence over time

Plant autofluorescence from photosystem II, termed delayed fluorescence (DF), can be used as a non-invasive, reporter-free method to monitor circadian rhythms in constant conditions [8]. One obstacle to imaging DF is that the signal is weak and transient, exponentially decaying after the plant is put into the dark [8]. Therefore, to capture DF, the imaging system must have the ability to rapidly switch from illumination to imaging in complete darkness with low background. We tested the ability of our imaging systems to monitor DF rhythms from wild-type and mutant Arabidopsis plants. In agreement with reported bioluminescent data from luciferase reporters, we were able to compare rhythms from the short period *toc1* mutant [14] and the arrhythmic *elf4* (*EARLY FLOWERING 4*) mutant [15] compared to wild-type plants (Fig. 7). Thus, these manually assembled imaging systems are capable of monitoring DF. This method should broadly apply to many plant species, as photosystem II is present in all land plants.

#### Fluorescence

**Fig. 8:**
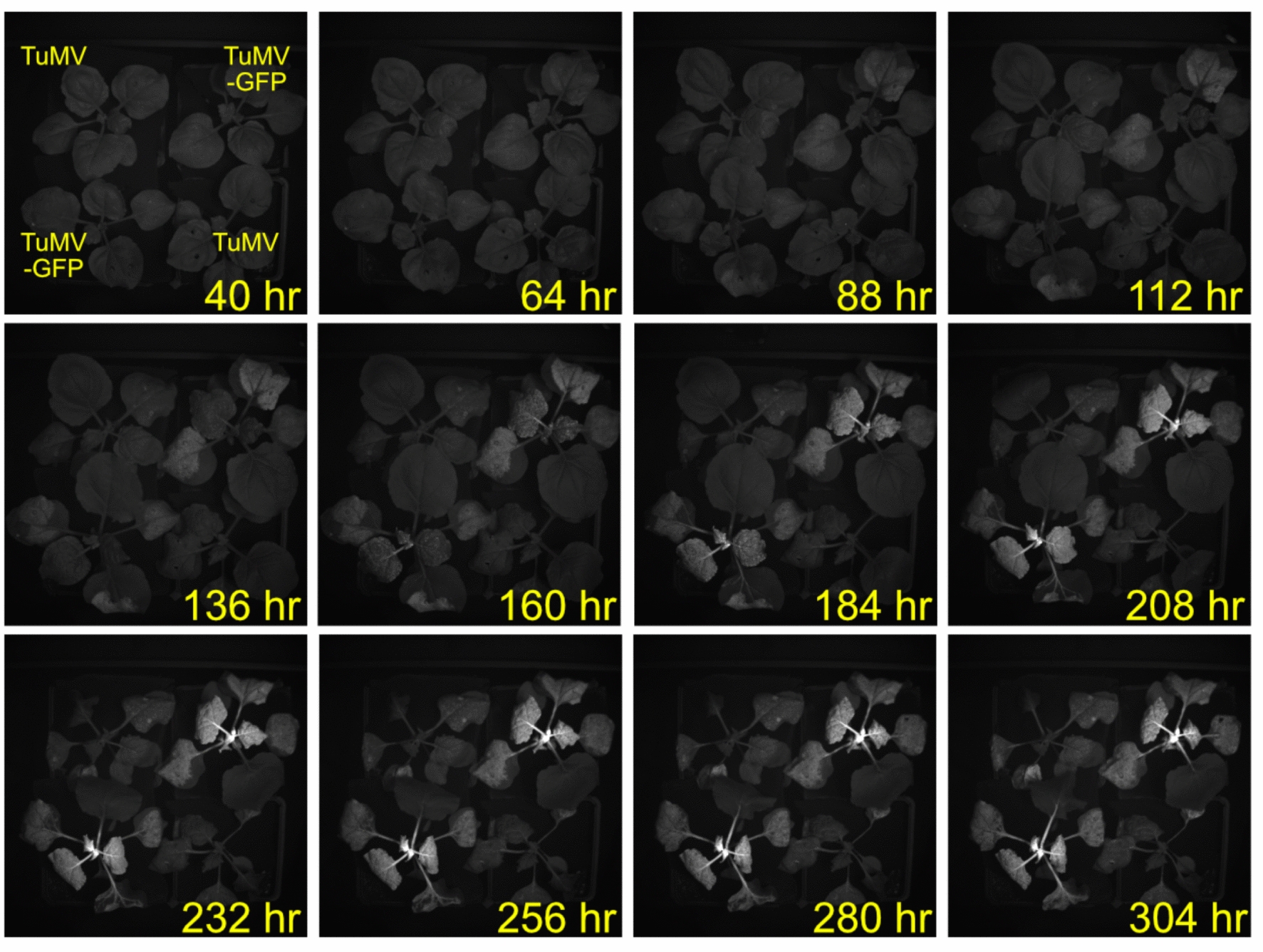
Imaging of infection and spread of Turnip Mosaic Virus (TuMV) expressing GFP in tobacco. *N.* *benthamiana* was subcutaneously inoculated with *A.* *tumefaciens* expressing either TuMV (top left and bottom right) or TuMV-GFP (Top right and bottom left). GFP signal was imaged with a narrow 532 nm bandpass filter during illumination with 400 nm light

Genetically encoded fluorescent reporters, such as GFP, are widely used reporters for monitoring diverse processes in a variety of organisms. For example, long-distance signaling in plants can be visualized through observation of reporter signal movement into organs outside of the originating tissue [16, 17]. We tested if our system could monitor virus infection and movement over a 2-week course of infection. We used Turnip Mosaic Virus (TuMV) or TuMV-GFP genomes delivered through agrobacterium transformation to infect the leaves of *N. benthamiana*. To visualize GFP fluorescence, the plants were illuminated with 400 nm light and imaged with a 532 nm narrow bandpass filter placed over the camera lens. We were able to observe both the local expression and spread of the TuMV-GFP at the site of inoculation and the spread of TuMV-GFP to newly formed leaves at later time-points (Fig. 8). Thus, our system can be used to monitor fluorescent reporters using the appropriate filter and illumination settings during imaging.

## Discussion

Luciferase reporters are a widely used method for tracking promoter activity in vivo, especially for studying the circadian clock [4, 5]. Circadian clock reporters are often used to assess the impact of gene mutations, exogenous treatments, or environmental perturbations on clock properties such as period, phase, and amplitude [9, 18–21]. Luciferase reporters can also be used to determine whether a gene is under circadian and/or diel control. A typical circadian experiment consists of a period of entrainment where the organism is grown under conditions with exogenous cues such as photo- or thermo- cycles. This entrainment acts as a synchronization signal to ensure that the endogenous oscillator is set to the same external rhythm, therefore reflecting the natural environmental conditions for that organism. Following entrainment, conditions are shifted to free-run where all exogenous cues are held constant (e.g. light and temperature). Any reporter that maintains a rhythmic pattern of activity under these free-run conditions is considered circadian. Many methods have been developed to assess rhythmicity, many of which are available in BioDare2 and described in Zielinski et al. [22]. For accurate estimates of circadian period and rhythmicity, it is recommended to have five complete cycles; however, it is possible to use three cycles, provided there is sufficient replication [22].

Depending on the lighting setup, images can be taken every one or two hours. If standard white LEDs are used that require a longer dark adaptation prior to imaging, a 2 h imaging design would be best to limit the dark period exposure on the plants. We find that 2 h imaging is sufficient for clock reporter assays to capture rhythmicity. If acute treatments are given, as shown in Fig. 5 with the pulse of red light, a 1 h image resolution may be required to detect altered reporter activity.

The use of single-wavelength LEDs provides additional advantages and flexibility to the system. The main advantage of this lighting system is the lack of phosphorescence once the lights are turned off, unlike white light LEDs with phosphor compounds that emit light long after the lights are turned off, requiring an extended dark period before imaging. Nonetheless, we demonstrated that white LEDs could be effectively used in this system as long as there is at least a ten-minute delay after lights-off before starting image capture (Fig. 1). Multi-channel wavelength LEDs provide control over the light spectrum, allowing users to test various lighting conditions and ratios depending on the experiment or plant being imaged. Lastly, the use of single-wavelength LEDs in combination with an ultrasensitive CCD camera facilitates the imaging of DF from plants. For circadian experiments, this is an extremely powerful technique for species without transgenic reporters. The newer model Heliospectra Dyna lights used in system #2 now have a white 5700 K LED channel that will phosphoresce and must be kept off at all times. Generally, a low amount of phosphorescence does not impact bioluminescent imaging, especially when a strong reporter is used. A non-transgenic wild-type plant is recommended to be used as a control to account for any background fluorescence.

For DF imaging, it is essential that all phosphorescence and any other light from outside the chamber be blocked out completely. Therefore, it is best to take a series of images with increasing exposure times in an empty chamber to test for external light sources that interfere with imaging experiments. To capture the DF, imaging must be taken immediately after the lights are shut off. If the lights are not equipped with remote access either through the plug-in described for system #1 or the Heliospectra software used in system #2, a USB-controlled power switch should be used to synchronize the lights and CCD camera with the computer clock.

In summary, we have described a flexible, controlled-environment imaging system for short and long-term monitoring of bioluminescence, DF, and fluorescence in several organisms. This system has the sensitivity, low background, and speed to detect low bioluminescent signals while possessing the dynamic range for compatibility with fluorescent imaging. The environmental chamber facilitates imaging experiments of dissociated cells like protoplasts to adult plants under a range of temperature, light, and humidity conditions. We have demonstrated the efficacy of this system for measuring bioluminescent reporters for circadian studies; however, this imaging system is widely applicable to other reporter-based experiments that require single or multiple time points. For example, we showed that this system could monitor pathogen infection spread in plant leaves over time, which could serve as a powerful, non-invasive method for studying plant–microbe interactions. Bioluminescent and fluorescent reporters have revolutionized our ability to perform non-invasive, multi-time point studies by eliminating the need to perform lengthy and complicated techniques such as RNA extraction with quantitative RT-PCR for potentially hundreds of samples. We anticipate that our flexible, user-friendly imaging system will accelerate scientific discovery by enabling more scientists to take advantage of these useful reporter systems.

## Methods

### System 1—Nusinow lab, Danforth center (Built 2015)

#### Chamber

Geneva Scientific, Model I-30NL, with a 2-inch access port for power and communication cord access. Black foam and blackout curtains around portholes prevented light from entering the chamber. All interior surfaces of the chamber, including shelves, walls, and inside of doors, were painted black with ChalkBoard Black paint (Rust-Oleum, Vernon Hills, IL). If the camera is mounted in the interior, one wire shelf will need wires in the middle removed to accommodate the lens from the camera and painted black (Fig. 2). Alternatively, a hole can be cut into the top of the chamber (either pre-ordered or cut by the user) to accommodate the lens and the camera mounted outside. A swatch of blackout curtain sandwiched between the camera and the exterior of the chamber was sufficient to block exterior light. The chamber should be in a darkroom so that images can be taken in total darkness without light leakage from the outside of the chamber. If a darkroom is unavailable, the chamber should be protected from external light using blackout cloth, black foam, or other light blocking methods.

#### Camera

Cooled, ultra-sensitive CCD camera, Pixis 1024B (Princeton Instruments, Trenton, NJ), backlit with anti-reflective coating. A custom 3D-printed mount was used to attach the camera to the wire rack or the top of the chamber (https://www.thingiverse.com/thing:432709).

#### Lens

The camera was outfitted with a Navitar NMV-25M1 (25.0 mm Focal length, f/1.4) lens, which can accommodate M35.5 × 0.5 filters for fluorescent applications. A narrow green bandpass filter (BN532-35.5, Midwest Optical Systems, Palatine, IL) was used for imaging Green Fluorescent Protein (GFP).

#### LED lights

Heliospectra lights (Model L1, Heliospectra LED lights, Göteborg, Sweden) with 7 customizable light wavelengths (400, 430, 450, 530, 630, 660, and 735 nm) were used. Outer housing was painted black with ChalkBoard Black paint (Rust-Oleum, Vernon Hills, IL) to minimize phosphorescence. The internal reflector surrounding the LEDs was also painted black. An internal green LED on the controller board inside the lights was also covered.

#### Computer

2.90 Ghz Intel i5 processor computer, Windows 7 OS, 64 bit (Dell). USB 2.0 minimum to communicate with the camera.

#### Software

µManager 1.4.22 software, windows 64-bit build (https://www.micro-manager.org/, Open Imaging, San Francisco, CA) [23, 24]. Concurrent control of the Heliospectra lights (running Firmware 2.2.25 and older ONLY) with a Pixis camera was managed by a custom µManager plug-in called LightsCameraAction (Compile plug-in from https://github.com/bryantdo/LightsCameraAction). The LightsCameraAction plug-in coordinates with the Heliospectra lights to ensure lights turn off during imaging. As the CCD camera is extremely sensitive to light, it is imperative that lights are turned off during image capture so as not to damage the sensitive camera. With the LightsCameraAction plug-in and accessory files properly placed into the correct folders in the µManager folder on the hard drive, micro-manager is ready to use with the Heliospectra lights (see github repository for instructions on how to compile the plug-in for use with µManager). Open µManager and load in a configuration for the camera that is compatible with imaging your sample (See details below). Then, open the LightsCameraAction plug-in, and input the IP address of the lights (lights should have a static IP address), the port 53610 (which is used for telnet-based communication), and a location to save images.

Set the desired number of images to acquire during the run. The system is set up with a running lighting schedule during the experiment. However, you can also begin an experiment without a schedule running by toggling off the required schedule running tab. Before starting an imaging run, one can test the lights by sending an on/off command or querying the available light wavelengths. By default, the imager is set to send an all lights off command to the lights so that it is dark during imaging. During fluorescence-based imaging experiments, you will replace this command with a "set wavelength" command that will maintain one or more wavelengths of light during imaging to excite a fluorescent molecule.

#### Heliospectra web interface

The Heliospectra lights are assigned a static IP address that can be used to navigate to a web interface control page. One can set the light intensity between an arbitrary value of 0–1000 for each wavelength (400, 430, 450, 530, 630, 660, and 735 nm). Diurnal light schedules can also be set using this interface; users can enter the time of day when lights should turn on and off or change intensity.

### System 2—Greenham lab, University of Minnesota, modeled after System 1 (built 2019)

#### Chamber

Binder model KFB720UL (Catalogue number 9020–0325) with a 50 mm access port for power and communication cord access was pre-ordered for the left side of the chamber. An additional access port on the right side may be warranted depending on the room setup and the width of cables for lights. To accommodate the camera lens, a 100 mm central-top access port was pre-ordered to keep the camera casing outside of the chamber. This is an essential consideration when using whole plants, as increased humidity levels will cause moisture damage to the camera. An eight-inch diameter circle in the top shelf was cut out, so the field of view was not obstructed (Fig. 3). Foil was wrapped around the exterior of the camera casing, the back of the environmental chamber, and around the entire exterior casing of Heliospectra lights. The BINDER KFB720UL also has a sealed interior door to avoid exterior light infiltration. If a dark room is not available, a door seal and additional foiling or blackout material around light sources are sufficient to prevent light contamination.

#### Camera

This system was tested with two CCD cameras.

1. Princeton Instruments Pixis 1024B_eXcelon (Princeton Instruments, Trenton, NJ), back-illuminated, AIMO CCD, 1024 × 1024 pixels. Dual speed readout, 2 MHz and 100 kHz 16 bit.
2. Oxford Instruments ANDOR iXon Ultra 888 (Oxford Instruments, Concord MA), back-illuminated EMCCD, 1024 × 1024, 30 MHz.

#### Lens

The camera is outfitted with a Navitar NMV-25M1 (25.0 mm Focal length, f/1.4) lens, which can accommodate M35.5 × 0.5 filters for fluorescent applications. For imaging Green Fluorescent Protein, a narrow green bandpass filter can be used (BN532-35.5, Midwest Optical Systems, Palatine, IL).

#### LED lights

Heliospectra Dyna-RX30 lights (Heliospectra LED lights, Göteborg, Sweden), 9 wavelength spectrum (380 nm, 400 nm, 420 nm, 450 nm, 520 nm, 630 nm, 660 nm, 735 nm, and 5700 K), 420 W power consumption and 85–265 VAC voltage range 50/60 Hz. Be sure to keep the 5700 K white LED off to avoid phosphorescence. An internal green LED light on the controller board inside the lights can be turned off in the "configuration" tab of each light's web interface.

#### Computer

A Windows PC running Windows 7 or later with at least 4 GB RAM and 256 GB of storage; examples of PCs successfully used for this task:

- Intel Core 2 Duo E8400 @ 3.00 GHz, Windows 7 64-bit, 8 GB RAM, 1 TB HDD
- 2.90 Ghz Intel i5 processor computer, Windows 7 OS, 64 bit (Dell). USB 2.0 minimum to communicate with the camera.

#### Network setup

To provide communication between µManager and the camera and a means of configuring the lights, a small computer network needs to be set up. Additionally, we had the requirement of remote upload of acquired images to cloud storage and a wirelessly-connected flood alarm to warn of leaks from the imaging chamber's water supply. This necessitated internet and wireless connectivity.

Internet connectivity was complicated by the lack of an Ethernet wall port in the imaging room. We set up a small local area network (LAN) using an Ethernet switch and a wireless travel router. We chose a router that supported the OpenWrt operating system so that the LAN could be connected to the university wireless network, which uses WPA2-Enterprise (PEAP-MSCHAPv2) security. Furthermore, the router's wireless capabilities were used to connect the flood alarm (Additional file 3: Figure S3).

#### Software

For newer Heliospectra lights running Firmware 3.0.0 and later, the free software provided by Heliospectra, System Assistant, is required.

### Driver installation and image configuration in µManager

Before images can be taken, the correct driver for the camera being used must be installed on the imaging computer, and the camera must be imported and configured in µManager. Software drivers are freely available from Princeton Instruments and Oxford Instruments for Pixis and Andor cameras, respectively. With Pixis cameras, install PICAM if you have a 64-bit system (https://www.princetoninstruments.com/products/software-family/pi-cam). PVCAM is the older equivalent for 32-bit (https://www.photometrics.com/support/software-and-drivers). For Andor cameras, the Andor Driver Pack (https://andor.oxinst.com/downloads/) must be installed in µManager's home directory.

Once the correct drivers have been installed, a configuration file for the camera in µManager must be created. This can be accomplished using the Hardware Configuration Wizard. Select the type of device (“PICAM” or “PVCAM” for PIXIS cameras; “Andor” for Andor) and accept the rest of the default settings. µManager should connect to the camera, which will be indicated by the absence of errors.

Open µManager and load the configuration you just created. The software should connect to the camera. µManager's interface, by default, consists of three main sections: on the upper left, there are controls for taking photos; on the upper right, a space for creating and selecting imaging settings, and an image histogram on the bottom. In the upper right area of the interface, click the “add group” button. A dialog will pop up asking you to name the group and select which settings you want to configure. Select “exposure,” “readout rate,” and “gain.” If you have an Andor camera, you will also want to select "output amplifier" and "timeout".

By creating the group, you are telling µManager what parameters you would like to configure. Actual values for these parameters can be saved in a variety of presets for that group. After creating the group, a dialog will appear asking you to create your first preset. Set the values you would like for the default, and then hit the “add new preset” button to create any additional presets. At a minimum, we recommend two presets, one for a live feed of the chamber for setup and taking reference images, and another for long exposures. A basic configuration for these two presets is provided below.

Note that, with Andor cameras, µManager might throw an error when using the lowest (100 kHz) readout rate. This is due to a programmed timeout that expires before the image can be transferred off the CCD. To fix this, simply set the “timeout” value in the preset to a larger value. We used 60,000. This is reflected in the example presets below:

#### Example µManager presets

PIXIS 1024B:

- Live:
  - Exposure: 10.
  - Gain: 2
  - Readout Rate: 2 MHz 16bit.
- Long Exposure:
  - Exposure: 60,000.
  - Gain: 1
  - Readout Rate: 100 kHz 16bit.

Andor iXon Ultra:

- Live:
  - Exposure: 10.
  - Output Amplifier: Electron Multiplying.
  - Pre-Amp Gain: Gain 2.
  - Readout Mode: 30.000 MHz.
  - TimeOut: 10,000.
- Long Exposure:
  - Exposure: 60,000.
  - Output Amplifier: Conventional.
  - Pre-Amp Gain: Gain 1.
  - Readout Mode: 0.100 MHz.
  - TimeOut: 60,000.

### Time-series acquisitions in µManager

µManager already provides tools for setting up a simple time series of evenly-spaced acquisitions through its “Multidimensional Acquisition” tool. However, this tool does not allow the user to set the start time of the series. Furthermore, it does not provide a method of taking multiple different time series simultaneously, such as when both delayed-fluorescence and luciferase imaging is desired in the same experiment. As such, we developed a Python script using the features of µManager 2.0.0 and the pycromanager Python library. It communicates with µManager through features introduced in µManager 2.0.0 to take a series of evenly-spaced images between two dates/times. Multiple time series can be acquired simultaneously by simply running multiple copies of the Python script with settings for each individual series. Documentation for installing and running the script is included in its README file on github (https://github.com/GreenhamLab/CCD_Imaging).

### Heliospectra light scheduling for late-model lights

Starting in firmware version 3.0.0, Heliospectra removed their publicly-documented protocol for controlling their ELIXIA, DYNA, and EOS lights remotely. This renders inoperable the “LightsCameraAction” µManager plug-in used in System 1. As such, the only method available for setting the light schedule was through the web interface for the Heliospectra lights. However, adding entries through this interface for turning the lights on and off repeatedly throughout the entire day was rather tedious. We developed a Python script that can automatically generate a configuration file describing a constant-light imaging schedule, which can then be read using the schedule import function on the Heliospectra web interface. Documentation for installing and running the script, as well as importing the schedule into the Heliospectra web interface can be found in the script's README file on github (https://github.com/GreenhamLab/CCD_Imaging).

### Plant growth (System 1)

Wild-type *CABpro::LUC*, *elf4-2 CABpro::LUC*, *toc1-4 CABpro::LUC*, and *elf3-1 elf4-3 CABpro::LUC* seedlings are in the Colombia background and were described previously [25, 26]. Arabidopsis seeds were surface sterilized in 20% bleach/0.1% triton-X100 for 5 min followed by three washes with sterile H_2_O. Seeds were plated on half-strength Murashige & Skoog (MS)-Agar (50% MS, 0.05% MES, 0.8% Agar, pH 5.7) with 1% (w/v) sucrose unless otherwise noted in square plates. After stratification at 4 °C for 2 days, plates were transferred to a Percival incubator (Percival-Scientific, Perry, IA) set to a constant temperature of 22 °C. Light entrainment was 12 h light/12 h dark (LD) cycles, with light supplied at 80 µmol m^−2^ s^−1^. *Nicotiana benthamiana* were grown on Berger 7–35% soil (Hummert, Earth City, MO) for 4–5 weeks at 22 °C, 16 h light/8 h dark cycles, 200 µmol m^−2^ s^−1^ before imaging. Cassava (*Manihot esculenta*), was grown at 28 °C, 12 h light/12 h dark cycles, 150 µmol m^−2^ s^−1^ before imaging. Light intensity was measured using a LI-COR LI-250A (Li-COR Biosciences, Lincoln, NE).

### Plant luciferase imaging for acute light responses (System 1)

Arabidopsis seeds were surface sterilized in 20% bleach/0.1% triton-X100 for 5 min followed by three washes with sterile H_2_O. ~ 25 seeds were plated into sterilized 1 cm long black straws inserted into square plates containing half-strength Murashige & Skoog (MS)-Agar (50% MS, 0.05% MES, 0.8% Agar, pH 5.7) with 1% (w/v) sucrose. After stratification at 4 °C for 3 days, plates were sprayed with 5 mM luciferin (Goldbio, Olivette, MO) prepared in 0.01% (v/v) Triton X-100 (Sigma-Aldrich) and exposed to white light for two hours to stimulate germination. After light treatment, plants were placed into the imager in constant darkness, 22 °C. 48 h later the seedlings were exposed to a single red-light pulse at 20 µmol m^−2^ s^−1^ for 10 min before continuing in constant darkness for the remainder of the experiment. 8 min exposures were used for imaging using a PIXIS 1024b camera.

### *Manihot esculenta* infection and imaging (System 1)

Bioluminescent *Xanthamonas phaseoli pv. manihotis* (Xam668) containing the pLUX plasmid was reported previously [2]. Leaves were inoculated with a solution of this strain (OD_600_ = 0.01), on either side of the major vein in two lobes. 48 h after inoculation, the leaf was detached and the lobes were separated and placed on plates containing 0.5 × Murashige and Skoog (MS) basal salt medium with 0.8% agar. The plates were transferred into the imaging chamber in 12 h:12 h light:dark, constant 28 °C under 40 µmol m^−2^ s^−1^ (measured with a LI-COR LI-250A, Li-COR Biosciences at plant level, Lincoln, NE), wavelengths 400, 450, 530, 630, 660, 735 nm set at intensity 950, 650, 400, 250, 150, and 1000, respectively (Heliospectra LED lights). 10-min exposures after a 3 min delay were used for imaging using a PIXIS 1024b camera.

### Delayed fluorescence imaging (System 1)

For delayed fluorescence imaging, seedlings were liquid sterilized and approximately 25 seed clusters were plated into square plates containing half-strength Murashige & Skoog (MS)-Agar (50% MS, 0.05% MES, 0.8% Agar, pH 5.7) with 1% (w/v) sucrose. The seedlings were stratified and entrained as above, then transferred into the imaging chamber after 6 days and into constant 22 °C, and constant light conditions at 70 µmol m^−2^ s^−1^ (measured with a LI-COR LI-250A, Li-COR Biosciences at plate level, Lincoln, NE), by setting wavelengths 400, 430, 450, 530, 630, and 660 nm to intensity 350 (Heliospectra LED lights). The delayed fluorescence was recorded for 6 days with an exposure time of 120 s after a 10 ms delay using a PIXIS 1024b camera [8].

### *N. benthamiana *TuMV infection and imaging (System 1)

Overnight saturated cultures of *Agrobacterium tumefaciens* strain GV3101 carrying either *pCB-TuMV* or *pCB-TuMV-GFP *[27] were diluted in 10 mM MgCl2 (OD600 = 0.8) and kept at room temperature for 1 ~ 2 h. The cultures were then spot-infiltrated into *N. benthamiana* from the abaxial side of leaves. After infiltration, the plants were transferred into the imaging chamber and grown in 12 h:12 h light:dark, constant 22 °C in 70 µmol m^−2^ s^−1^ (measured with a LI-COR LI-250A at plant level, Li-COR Biosciences, Lincoln, NE), wavelengths 400, 430, 450, 530, 630, and 660 nm set at intensity 350 (Heliospectra LED lights). The plants were imaged for GFP fluorescence every 60 min after a 10 s delay for 400 ms under 400 nm light at an intensity of 600 (6.2 µmol m^−2^ s^−1^, Heliospectra LED lights). GFP imaging requires a narrow green bandpass filter to be outfitted over the camera lens (BN532-35.5, Midwest Optical Systems, Palatine, IL).

### Luciferase imaging of Arabidopsis seedlings in Growfilm-fitted Binder chamber (System 2)

Wild-type *CCA1pro::LUC* seedlings are in the Columbia background. Seeds were surface-sterilized with 20% bleach solution, stratified in 4 °C in the dark for 2 days, then sown on half-strength MS-Agar (50% MS, 1% sucrose, 0.05% MES, 0.8% Agar, pH 5.7) in square plates. Plates were placed in a Precision Plant Growth Chamber (Thermo Scientific, Waltham, MA) under a 12 h:12 h light:dark (LD) cycle and 20 °C:16 °C hot:cold (HC) cycle. Ten days after sowing (DAS), select individual seedlings were transferred to each well of a 48-well plate (Genesee Scientific, San Diego, CA, Ca. No. 25–103), containing 500 µL of solidified half-strength MS-Agar per well. 5 mM luciferin was sprayed over the seedlings. Alternatively, 40 µL of 5 mM luciferin can be pipetted into each well and gently nutated by hand to spread the luciferin. To limit condensation build-up during imaging, two full pumps of thick foamy hand soap (Clean Revolution Foaming Hand Soap) was dispensed on the lid of the plate and rubbed dry using a paper towel until the soap disappeared. The lid was then allowed to dry under the laminar flow hood for 5 min. Alternatively, a plate seal (Genesee Scientific, San Diego, CA, Ca. No. 12–167) can be applied to the plate and holes punched into each well using a syringe needle.

To image, seedlings were immediately transferred to continuous light in a Binder chamber (model KFB720UL) outfitted with a pair of Growfilm White Lighting sheets (Heilux LLC, Eden Prairie, MN) connected to a manual timer (in place of Heliospectra lights). Images were acquired every 2 h. Growfilms (PPFD ~ 150 µmol m^−2^ s^−1^, measured with a Sun System PAR Meter at plate level) were turned off for 15 min, as the manual timer can only be manipulated in 15-min increments. The image was acquired at minute 10 (with a 4 min exposure), then lights turned on at minute 16. The image was not acquired until minute 10, so that residual phosphorescence would not be captured. During imaging, Binder conditions were set to 20 °C constant temperature and 40% constant relative humidity (RH). Image analysis was performed using Fiji and BioDare2.

### Temperature cycle entrainment and luciferase imaging of Arabidopsis seedlings in Heliospectra-fitted Binder chamber (System 2)

As done for seedlings imaged in the Growfilm-fitted Binder chamber, Seeds were surface-sterilized, stratified, sown on half-strength MS-Agar (1% suc) in square plates, then seedlings 10 DAS (grown under a 12 h:12 h LD cycle in a growth room, with an ambient temperature of 19.5–23 °C and ambient RH of 26.5–36.5%) were transferred to a 48-well plate containing half-strength MS-Agar (1% suc). Then, 40 µL of 5 mM luciferin was pipetted into each well and seedlings were transferred to the Binder imaging chamber outfitted with Heliospectra lights for a 5-day step temperature cycle entrainment (12 h 22 °C:12 h 12 °C and constant light with PPFD ~ 120 µmol m^−2^ s^−1^, ~ 25% RH) or a 5-day ramp temperature cycle (12 h ramping up to 22 °C with 1-2˚C/h increments and 12 h ramping down to 12 °C with 1˚C/h increments PPFD ~ 120 µmol m^−2^ s^−1^ measured with a Sun System PAR Meter at plate level, ~ 25% RH). Heliospectra wavelengths 380, 400, 420, 450, 530, 620, 660, and 735 nm were selected (5700 K was not selected). Data Loggers were placed by the plates in Binder to record actual temperature and RH. Luciferin was reapplied to temperature cycle-entrained seedlings, then released into free-running conditions (constant 22 °C, constant light PPFD ~ 120 µmol m^−2^ s^−1^, ~ 25% RH) for 5 days. During imaging every 2 h, Heliospectra lights are turned off for 7 min. An image was acquired at minute 4 (with a 3 min exposure), then the lights turned on at minute 8. The image was not acquired until minute 4 so residual phosphorescence would not be captured.

### Image analysis

#### Analysis of luciferase activity using Metamorph and BioDare2

Image stacks were imported into the Metamorph imaging software as outlined previously [28] (Molecular Devices, Sunnyvale, CA) by selecting "File" > "Open Special" > "Build Stack" > "Numbered Names". Once images have loaded, adjust the contrast and false coloring as needed to best visualize the plants by adjusting the "A 10" slider. Select an image where the plants are at their largest and can easily be seen. Use this image to draw regions.

Right-click over the “Region Tool Bar” and open the “Region Tool Properties”. Set "Region Size" to encircle the size of the entirety of your plants at their largest, i.e., 20 × 20 for small plants or 40 × 40 for large plants. Choose the "Ellipse Region" (circle shape) from the Regions Tool Bar and click on the center of each plant to draw regions around all plants to be analyzed. Save regions by selecting "Regions" > "Save Regions". This way, you can load regions to this image stack in the future to check which regions are assigned to which plant. The resulting stack of images was processed by Metamorph imaging software (Molecular Devices, Sunnyvale, CA). Three to five regions from areas of the plate where no plants are present were used for background.

Before exporting region data, close any open Excel files. Select "Log" > "Open Data Log" and name the sheet with an appropriate name. Select "Apps" > "Graph Intensities" (if this option is not there, you need to import the Graph Intensities app from the Metamorph website). Choose "Stack" for the "Measure From" section. Select "Plane Number" for the "Measure Regions Over" section. Select "Integrated" in the "Region Measurement" section. Under "Configure Log" check: "Image Name", "Image Plane", "Image Data & Time", "Region Name", and "Integrated". Click "OK" and then "Begin". Metamorph will begin recording the intensity data from all regions into the opened Data Log Excel file. Save the resulting Excel file for downstream circadian analysis. Signal from background regions was averaged and subtracted from seedling signal to provide a background subtracted value. Background-subtracted seedling rhythms were plotted and analyzed using Biodare 2 [22]. For period analysis in BioDare2, "linear dtr" was selected for detrending, 18–34 h was selected for expected period, and "FFT NLLS" was selected for the analysis method. A detailed step-by-step protocol is provided (Additional file 1).

#### Image analysis of luciferase activity using Fiji and BioDare2

Before acquiring time-course images, a reference image was taken in which the wells of the plate were clearly visible. This reference image (16-bit type) was opened in Fiji (https://imagej.net/software/fiji/) open software (version 2.1.0/1.53C) on a Mac OS (version 11.3.1). Once the reference image was opened, brightness/contrast was adjusted as necessary to see the wells more clearly by clicking "Image" > "Adjust" > "Brightness/Contrast…". The "oval" tool was selected on the tool bar, then pressing the "shift" key, a perfect circle was traced around a single well. This circle was then added to the "ROI Manager" window by clicking "Edit" > "Selection" > "Add to Manager". This circle can also be saved as a region of interest (ROI) by clicking "File" > "Save As" > "Selection" > "Region of Interest…". An ROI set was created to measure bioluminescence in multiple wells. To build an ROI set, the previously saved ROI in the "ROI Manager" window was highlighted. Of note, the "Show All" and "Labels" boxes in the "ROI Manager" window were unchecked, which we found to be critical. The highlighted circle was moved to another well, then this region was added to the "ROI Manager" window. This was repeated until all wells of interest were added to the ROI Manager. In addition, the highlighted circle was also moved to a spot on the image that was "black" and empty to capture the background signal. This ROI set was then saved as a .zip file by clicking "More" > "Save…". To use this ROI set again, open the .zip file in Fiji (do not unzip).

Bioluminescence measurements were set in Fiji by clicking "Analyze" > "Set Measurements…", then in the "Set Measurements" window, the "Mean gray value" box was checked, then the "Ok" button was pressed. The folder containing the images was opened in Fiji, and when prompted, the "Convert to RGB" and "Virtual Stack" boxes were left *unchecked*. We found that the background intensified when images were converted to RGB. The ROI set was then overlaid on the image stack by checking "Show All" in ROI Manager. To measure the first image in the stack, the "Measure" button was pressed in ROI Manager. The same was repeated in the following image until all images in the stack were measured. The raw values in the "Results" window were copied and pasted into an Excel spreadsheet, which was then reformatted for input into BioDare2 for rhythm analysis. The Excel spreadsheet format was such that replicate seedlings were arranged in columns and ZTs were in rows (see the publicly accessible file in Biodare2). The "mean gray value" of the background for each ZT was subtracted from all the "mean gray values" of the replicates in that ZT. Then each replicate was averaged over time to produce an average value. All the 'mean gray values' for each replicate were normalized to that average value. The data with normalized replicates were uploaded into BioDare2. See figure legends for specific parameters selected in BioDare2. For period analysis in BioDare2, "linear dtr" was selected for detrending, 18–34 h was selected for expected period, and "FFT NLLS" or "MESA" was selected for the analysis method. A detailed step-by-step protocol is provided (Additional file 2) with sample data available on github https://github.com/GreenhamLab/CCD_Imaging.

## Supplementary Information

**Additional file 1. **Detailed step-by-step protocol for luciferase image analysis using Metamorph software and formatting for upload to BioDare2.

**Additional file 2. **Detailed step-by-step protocol for luciferase image analysis using Fiji software and formatting for upload to BioDare2.

**Additional file 3.** Supplemental Figures 1–3.

## Data Availability

Code is available at https://github.com/bryantdo/LightsCameraAction and https://github.com/GreenhamLab/CCD_Imaging
