# Supplementary material for: Assembly and operation of an imaging system for long-term monitoring of bioluminescent and fluorescent reporters in plants
Source: Plant Methods. 2023 Mar 1;19:19. doi: 10.1186/s13007-023-00997-0 (PMC9976486; doi:10.1186/s13007-023-00997-0)
Supplement: Supplementary file 1 — Additional file 1. Detailed step-by-step protocol for luciferase image analysis using Metamorph software and formatting for upload to BioDare2. [file 13007_2023_997_MOESM1_ESM.pdf]

# MetaMorph Analysis Protocol

## Metamorph

### Import Images

1. **Start with a series of .tif images** saved over the course of a run in the imaging chamber
2. **Open MetaMorph** Select File -> Open Special -> **Build Stack** -> Numbered Names
3. Select **the first image** and **last images** and click  -> OK.
4. **Click** the **bottom icon** on the **side "A 10"** and choose the option that best suits the images, taking note of how the light bar changes. Uncheck "auto scale" to apply the selected setting to all images in the stack
    a. NOTE: No matter which settings are selected for viewing, the exported intensity values will remain the same.
5. **Adjust brightness** using the slide bars on the left & **choose an image** that shows each plant as clearly as possible

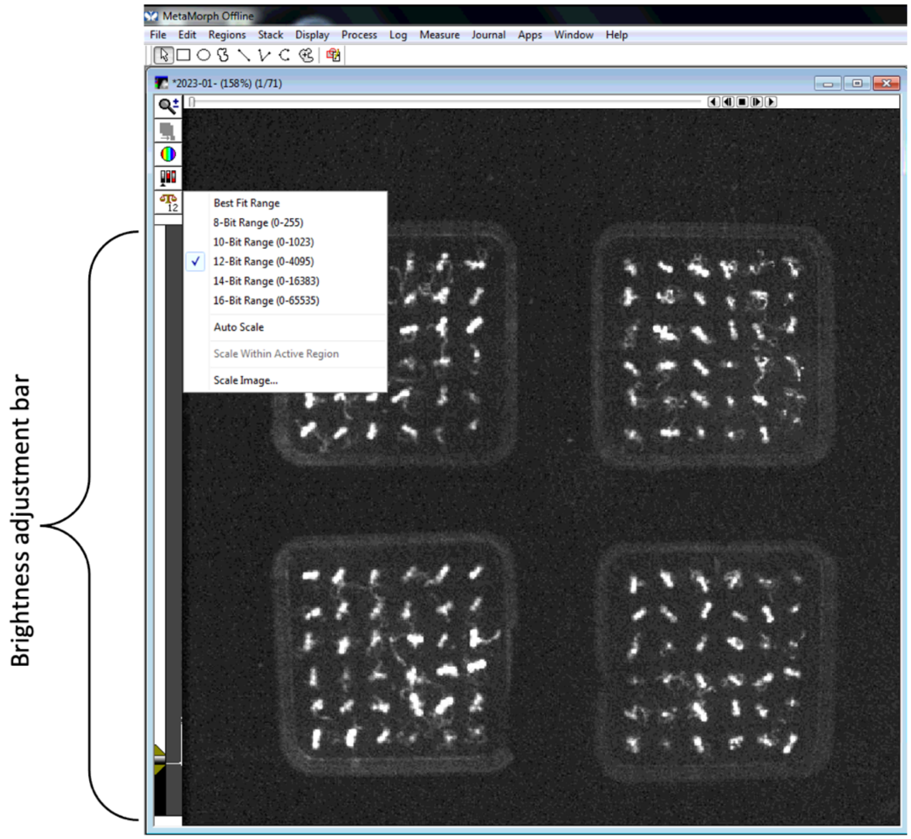


### Draw Regions

1. Check the **Region Tool Properties** on end of Regions Tool Bar – Typically check use **same color** & **lock region** size
2. **Set Region size** appropriate to the size of the plants
3. Choose **Ellipse Region** (Circle Shape) from Regions Tool Bar
4. **Draw Regions** around all plants to be analyzed, keeping genotypes grouped by numerical order
5. On each plate, **draw a region** that does not overlap with any plants to serve as background for downstream normalization

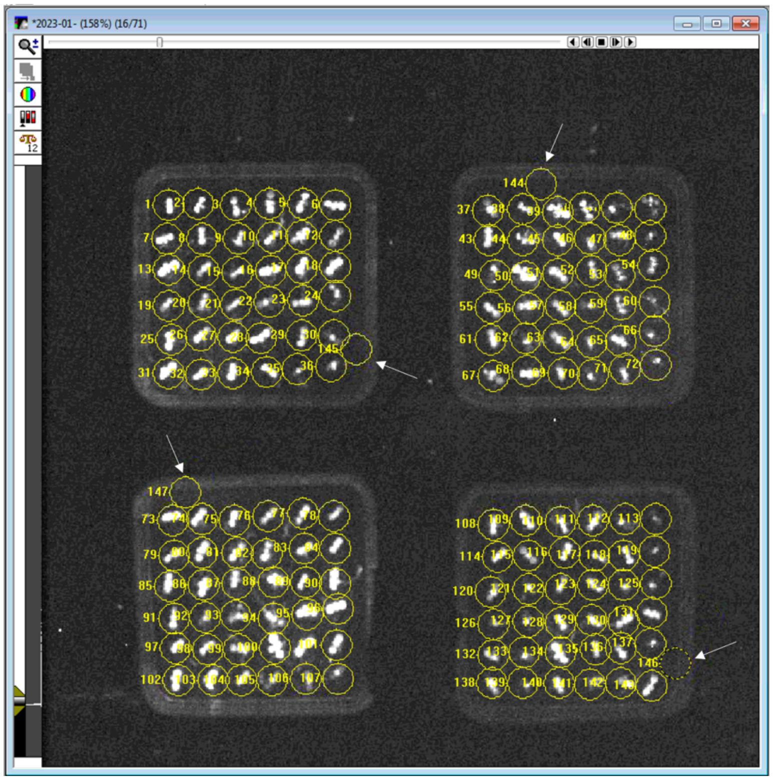


6. After drawing, **switch to** the **locator tool** (Arrow) and make sure all **circles** are **centered** on each plant
7. **Click through image stack** to ensure that regions adequately cover each plant over the entire course of imaging
8. Regions -> **Save Regions** file

## Export Intensities

1. **Close** any open **Excel** files.
2. Select Log -> **Open Data Log**
3. Select Dynamic Data Exchange (DDE) and click OK
4. Set the application to Microsoft Excel, name the sheet, and ensure starting row and column are both “1”. Click OK.

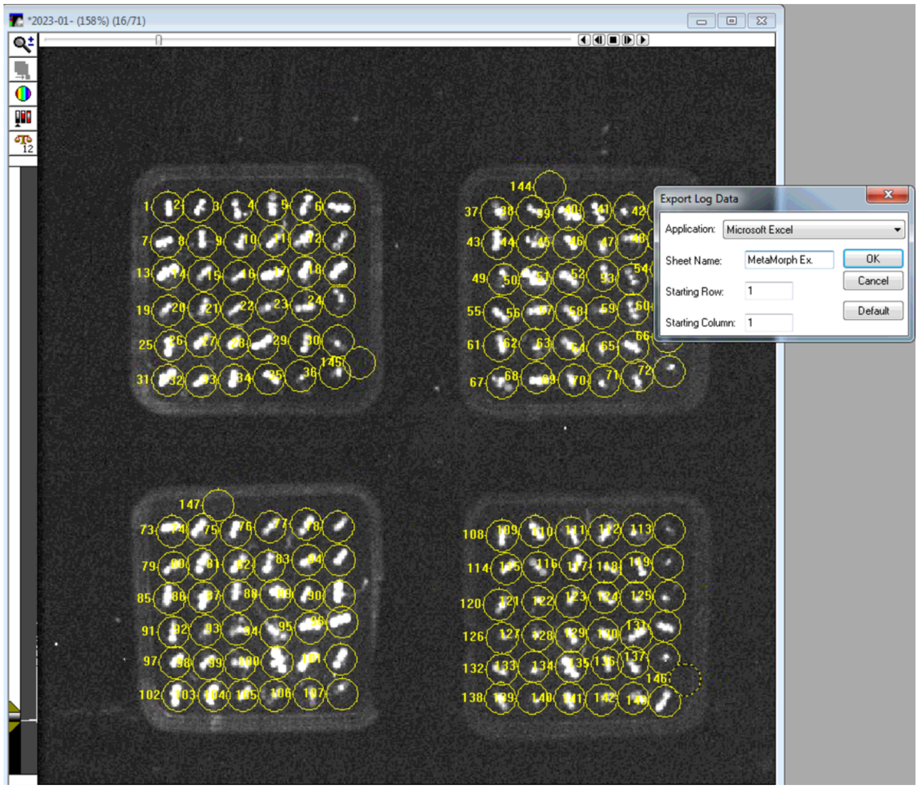


5. Select Apps -> **Graph Intensities**
6. Choose **Stack** in Measure From
7. Choose **Plane Number** in Measure Regions Over
8. Choose **Integrated** in Region Measurement
9. Under **Configure Log** select: “Integrated”. Click OK

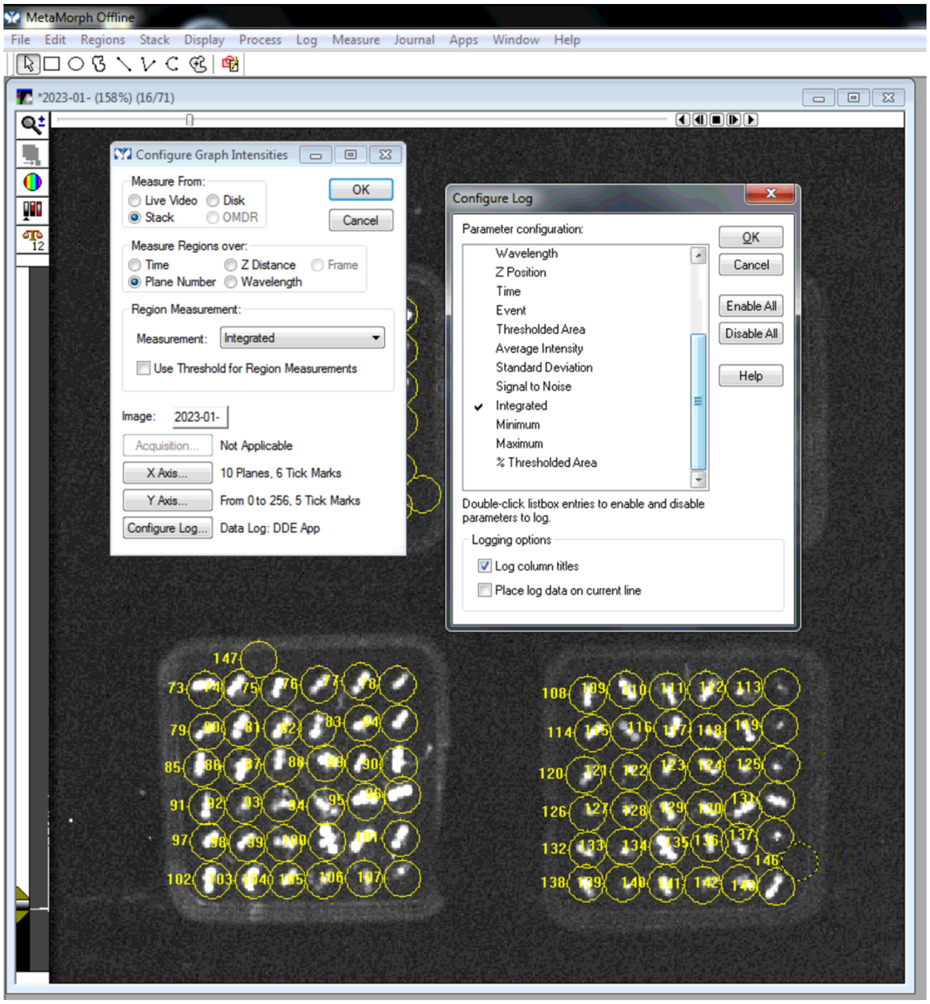


10. Click OK & **Begin**
11. When finished, **save** Excel File

## Preparing Excel File for BioDare

1. **Insert** a new column and **average the background intensities** for each background region across all images

| EN | EO | EP | EQ | ER | ES | ET |
|---|---|---|---|---|---|---|
| ROI '142' E | ROI '143' E | BG 1 | BG 2 | BG 3 | BG 4 | AVG BG |
| 148070 | 149173 | 145423 | 145218 | 145805 | 145874 | 145580 |
| 149173 | 149291 | 145854 | 145714 | 146126 | 146441 | 146033.8 |
| 148668 | 149284 | 145306 | 145013 | 145534 | 145990 | 145460.8 |
| 148918 | 150301 | 145151 | 144636 | 145291 | 145726 | 145201 |
| 148658 | 149791 | 145389 | 144891 | 145425 | 145737 | 145360.5 |
| 148916 | 149747 | 145839 | 145462 | 145883 | 146324 | 145877 |
| 149170 | 149838 | 145833 | 145438 | 145965 | 146424 | 145915 |
| 149253 | 149898 | 145587 | 145273 | 145794 | 146418 | 145768 |
| 149556 | 150170 | 145788 | 145529 | 145982 | 146683 | 145995.5 |
| 149580 | 150272 | 145766 | 145524 | 146375 | 146693 | 146089.5 |
| 149618 | 150398 | 145778 | 145598 | 146062 | 146652 | 146022.5 |
| 149733 | 150386 | 145757 | 145557 | 146022 | 146658 | 145998.5 |
| 150038 | 150369 | 146039 | 145796 | 146370 | 146785 | 146247.5 |
| 150070 | 150410 | 146206 | 146060 | 146503 | 147154 | 146480.8 |
| 149946 | 150412 | 146037 | 145947 | 146399 | 146877 | 146315 |
| 150130 | 150510 | 146176 | 146065 | 146577 | 147033 | 146462.8 |

2. **Open** a new sheet, rename this sheet “Background subtracted intensities,” and **paste** the averaged background **values** into the first column
3. **Copy** the ROI headers for **each sample (not background regions)** and **paste** into Background subtracted intensities sheet

4. **Subtract the average background** from each sample intensity by **using the formula** "='Sheet 1'!A2-'Sheet 2'!$A2"
    a. Note: Make sure to include the $ to lock the column for background intensities

B2 | fx ='MetaMorph Ex.'!A2-'BG subtracted intensities'!$A2

| | A | B | C | D | E | F | G | H | I |
|---|---|---|---|---|---|---|---|---|---|
| 1 | AVG BG | ROI '1' Elli | ROI '2' Elli | ROI '3' Elli | ROI '4' Elli | ROI '5' Elli | ROI '6' Elli | ROI '7' Elli | ROI '8' Elli |
| 2 | 145580 | 2311 | | | | | | | |
| 3 | 146033.8 | | | | | | | | |
| 4 | 145460.8 | | | | | | | | |
| 5 | 145201 | | | | | | | | |
| 6 | 145360.5 | | | | | | | | |
| 7 | 145877 | | | | | | | | |
| 8 | 145915 | | | | | | | | |
| 9 | 145768 | | | | | | | | |
| 10 | 145995.5 | | | | | | | | |
| 11 | 146089.5 | | | | | | | | |
| 12 | 146022.5 | | | | | | | | |
| 13 | 145998.5 | | | | | | | | |
| 14 | 146247.5 | | | | | | | | |
| 15 | 146480.8 | | | | | | | | |
| 16 | 146315 | | | | | | | | |
| 17 | 146462.8 | | | | | | | | |

5. **Apply** this formula to all cells
6. **Open** a new excel workbook and paste the **values** for each ROI, beginning in **column B**
7. In **column A,** input the time each image was taken
8. Edit the name of each ROI to reflect the name of its corresponding sample

A1 | fx Circadian Time (CT)

| | A | B | C | D | E | F | G | H | I |
|---|---|---|---|---|---|---|---|---|---|
| 1 | Circadian Time (CT) | CCA1::luc | CCA1::luc | CCA1::luc | CCA1::luc | CCA1::luc | CCA1::luc | CCA1::luc | CCA1::luc |
| 2 | 0 | 2311 | 1552 | 1261 | 1566 | 2460 | 2912 | 2640 | 1278 |
| 3 | 1 | 4091.25 | 2773.25 | 2129.25 | 1472.25 | 2319.25 | 2803.25 | 2810.25 | 1805.25 |
| 4 | 2 | 4800.25 | 4163.25 | 2442.25 | 1855.25 | 2930.25 | 3679.25 | 3657.25 | 2308.25 |
| 5 | 3 | 4712 | 3889 | 3429 | 2982 | 4328 | 5442 | 4848 | 2928 |
| 6 | 4 | 4023.5 | 3021.5 | 2421.5 | 2391.5 | 3527.5 | 4157.5 | 3759.5 | 2092.5 |
| 7 | 5 | 4727 | 2856 | 2440 | 2467 | 3534 | 3968 | 4138 | 1978 |
| 8 | 6 | 5316 | 3231 | 2389 | 2585 | 3438 | 4085 | 4244 | 2248 |
| 9 | 7 | 5512 | 3654 | 2587 | 2841 | 3721 | 4154 | 4501 | 2511 |
| 10 | 8 | 5686.5 | 3757.5 | 2753.5 | 3209.5 | 4140.5 | 4586.5 | 4844.5 | 2825.5 |
| 11 | 9 | 5730.5 | 3553.5 | 2863.5 | 3434.5 | 4377.5 | 4811.5 | 5070.5 | 3769.5 |
| 12 | 10 | 5784.5 | 3621.5 | 2955.5 | 3809.5 | 4666.5 | 5523.5 | 5223.5 | 3133.5 |
| 13 | 11 | 5897.5 | 3810.5 | 3083.5 | 3970.5 | 4879.5 | 5876.5 | 5462.5 | 3240.5 |
| 14 | 12 | 6060.5 | 3877.5 | 3081.5 | 4201.5 | 4858.5 | 6123.5 | 5425.5 | 3402.5 |
| 15 | 13 | 6018.25 | 3735.25 | 2916.25 | 4041.25 | 4625.25 | 5875.25 | 5360.25 | 3074.25 |
| 16 | 14 | 5810 | 3866 | 2949 | 4008 | 4490 | 5902 | 5254 | 3386 |
| 17 | 15 | 5724.25 | 3859.25 | 2870.25 | 3819.25 | 4495.25 | 5781.25 | 5057.25 | 3322.25 |

9. Save excel sheet and proceed with analysis in BioDare
