# Supplementary material for: Assembly and operation of an imaging system for long-term monitoring of bioluminescent and fluorescent reporters in plants
Source: Plant Methods. 2023 Mar 1;19:19. doi: 10.1186/s13007-023-00997-0 (PMC9976486; doi:10.1186/s13007-023-00997-0)
Supplement: Supplementary file 2 — Additional file 2. Detailed step-by-step protocol for luciferase image analysis using Fiji software and formatting for upload to BioDare2. [file 13007_2023_997_MOESM2_ESM.pdf]

LUC Image Analysis in Fiji

FIJI

CRITICAL: The images must be 16-bit type.

1. Open reference image on Fiji (ImageJ). Adjust brightness by going to Image → Adjust → Brightness/Contrast.

2. Select rectangle or oval tool on Fiji, depending on whether you have plates or wells, and select your "model" region of interest. Hit "Command + T". This will add your selection to your "ROI Manager".
CRITICAL: You must do this on an image that is not part of the stack, else when you measure, your values will all be the same.

3. Save your "model" region of interest by hitting File > Save As > Selection

4. In your ROI Manager, highlight your first selection. Your box or circle will also be highlighted. **CRITICAL:** Make sure the "Show All" and "Labels" boxes are *unchecked*.

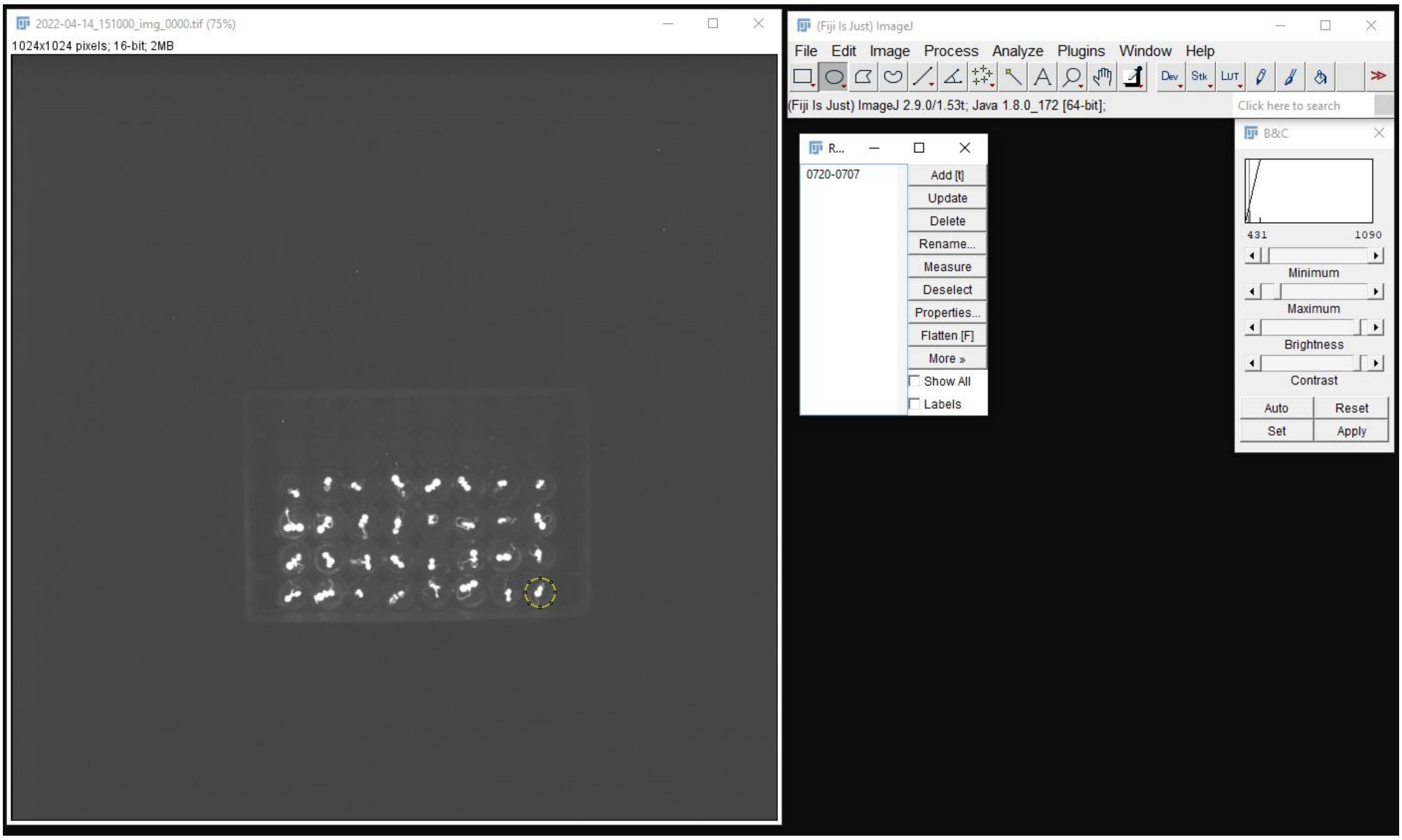

Selection of initial ROI

5. Move your box or circle to the next region of interest. Hit "Command + T" again.

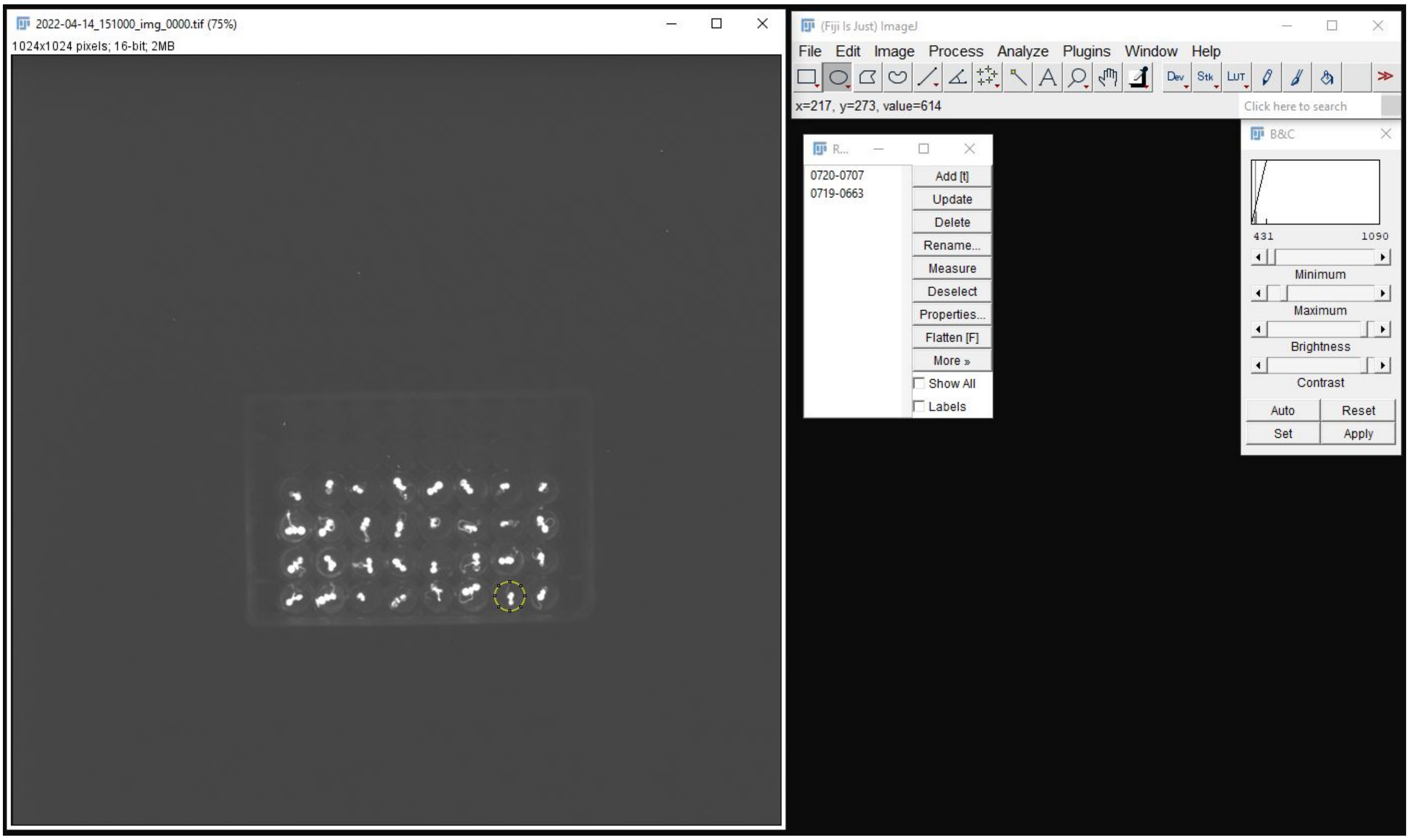

Moving the ROI circle to choose the next ROI

6. Repeat this until all your regions of interest are selected.

IMPORTANT: Make sure that your ROIs are in order and all grouped within one genotype!

NOTE: In addition to choosing all regions of interest, it is a good idea to also select 3-5 "blank" spaces (empty wells, empty pots or even empty space in the image). During analysis, these values can be averaged and subtracted from the experimental ROIs to control for background noise. **CRITICAL**: Select these regions last so that they are always the last measurements taken for each image.

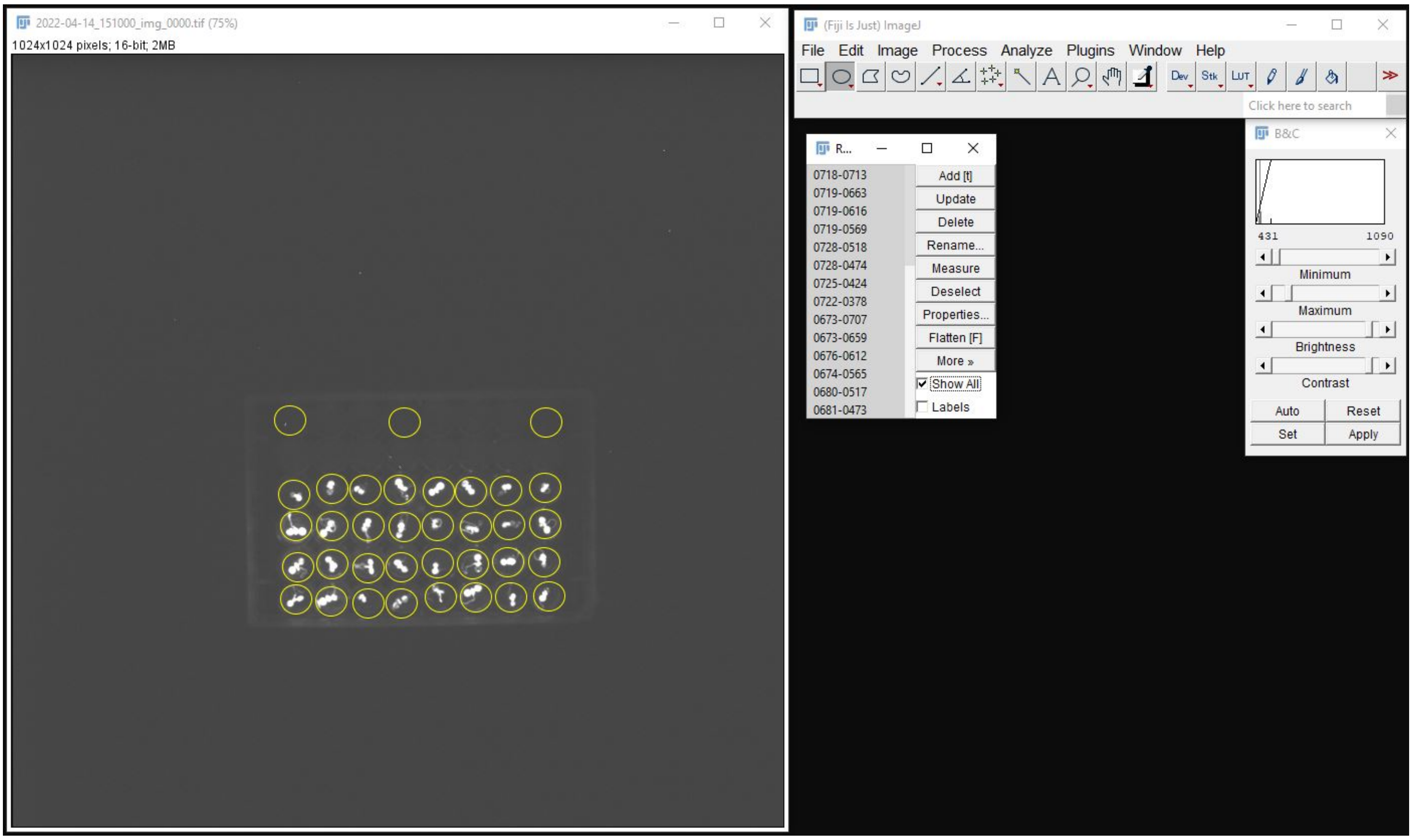

Reference image with all ROIs selected - 32 wells containing plants, and three blank wells used to correct for background noise.

7. Open your LUC stack image in Fiji by dragging the folder containing your image sequence to the ImageJ bar. (**CRITICAL:** When prompted, leave "Covert to RGB" and "Virtual Stack" boxes *unchecked*.)

8. Overlay your ROIs by checking "Show All" and check to see that all your selections fit your LUC signals. If you need to adjust some of them, highlight the ROI in the ROI Manager, move your box or circle, hit "Command + T", then delete the original. **IMPORTANT**: Doing this actually puts them out of order!

9. To save your ROI set, highlight all and hit "More > Save"

10. Go to "Analyze" in ImageJ and select "Set Measurements". In the pop-up window, select only "Mean Gray Value".

11. To measure, highlight all and hit "Measure" or "Control + M" on the ROI Manager. Switch to the next image using the mouse scroll.

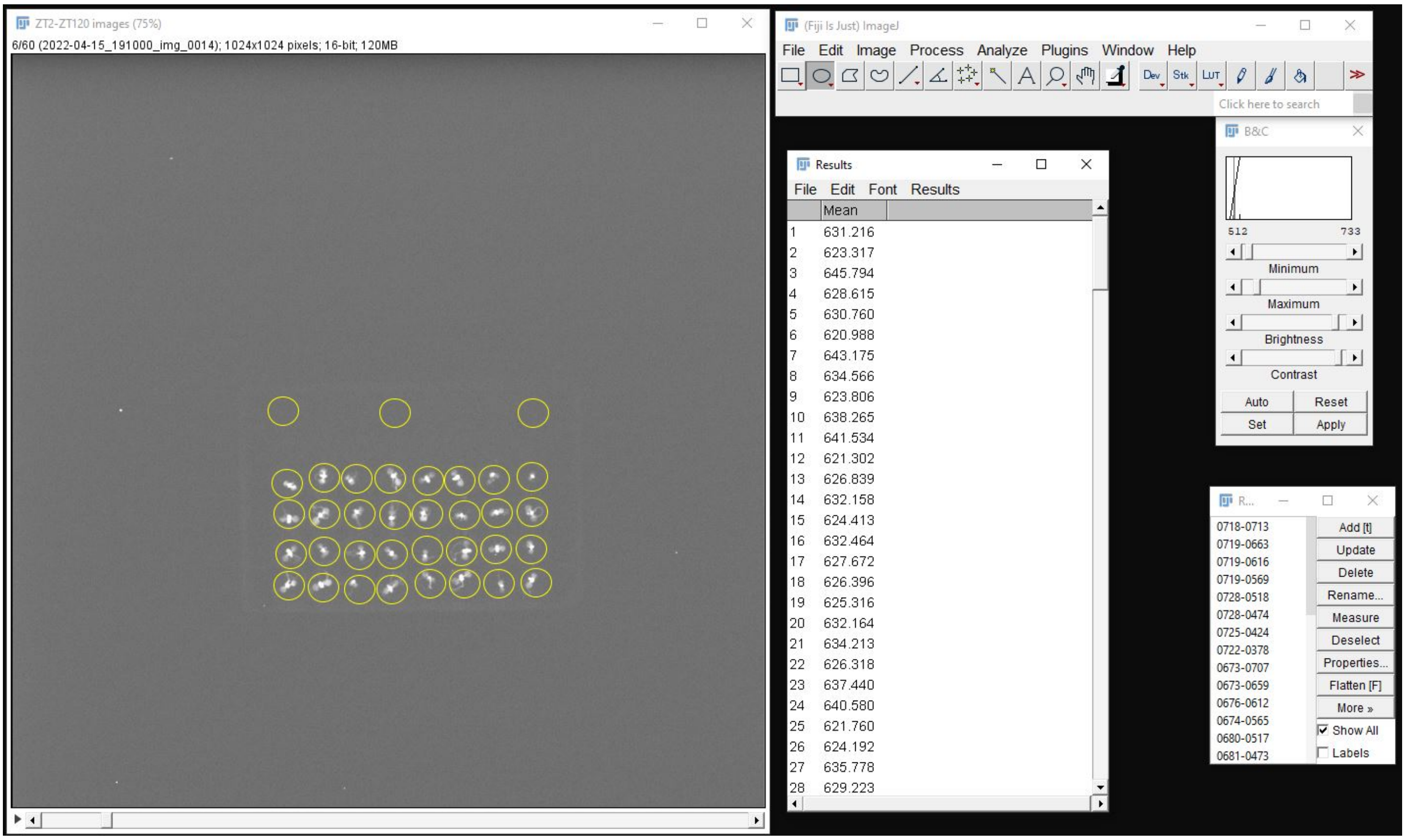

Results after applying the ROIs to the image stack and getting mean gray value for each ROI at each image.

ADDITIONAL NOTES

When setting up for imaging, make sure that the plates are not too far from the center of the camera lens.

If using a strong reporter (eg: Arabidopsis *CCA1p:LUC*) and long exposure, note that signal from one well will bleed in to nearby wells. It is important to use either black plates or to use adequate spacing around plants. It is best not to image a strong and weak reporter together.

ARRANGING DATA FOR IMPORT INTO BIODARE2

NOTE: There are multiple ways to accomplish this. We have provided versions for Excel and R.

CRITICAL: It is essential that you know exactly which ROI corresponds to each replicate. It is possible to rename the ROIs if that makes it easier to keep track of them. The order of the data output by Fiji will correspond to the order of ROIs. Save a backup of the raw data before proceeding with any analyses.

EXCEL:
Copy the two columns of data from Fiji (the measurement serial number and the measurement (i.e. mean gray value)). Paste these into an Excel Spreadsheet.

In a new column (other than column C)), use the following formula:
=INDEX($B$1:BX,ROW(C1)+(Y*(COLUMNS($C$1:C$1)-1)))

where X is the last measurement serial number (for example, if you have 56 total measurements, this would be 56) and Y is the sample size of your experiment (for example if you measured 7 plants, this would be 7)

Critical: You must lock the data column in place using the dollar signs!

For the provided sample dataset, there are 32 plants and 3 blank wells, for a total of 35 observations per time point, and 60 total time points for a total of 2100 observations. Thus, the formula used is:

=INDEX($B$1:$B$2100,ROW(C1)+(35*(COLUMNS($C$1:C$1)-1)))

Extend this formula into a grid where the number of rows is the sample size (ie, Y) and the number of columns is equal to the number of images you are analyzing. (Hint: an easy way to do this is to set up a grid beforehand by changing the color and using a guiding row/column and fill in the spots).

The data will be arranged into a grid.

D1 =INDEX($B$1:$B$2100,ROW(C1)+(35*(COLUMNS($C$1:C$1)-1)))

	A	B	C	D	E	F	G	H	I	J	K
1	1	631.216		631.216	635.509	635.955	634.256	629.087	626.782	627.304	623.294
2	2	623.317		623.317	626.648	625.756	623.651	619.821	618.792	619.927	616.223
3	3	645.794		645.794	653.463	651.262	643.889	635.124	630.966	629.383	623.698
4	4	628.615		628.615	629.908	628.556	626.056	622.779	621.762	622.802	618.441
5	5	630.76		630.76	637.443	638.851	635.565	628.682	626.28	627.029	622.894
6	6	620.988		620.988	624.235	623.9	622.372	618.858	618.24	619.76	616.209
7	7	643.175		643.175	648.162	644.684	640.36	634.053	631.197	630.158	624.464
8	8	634.566		634.566	639.494	638.363	635.134	630.438	629.333	629.852	625.481
9	9	623.806		623.806	627.815	627.312	626.237	622.669	621.553	622.404	618.457
10	10	638.265		638.265	642.281	639.628	635.267	628.98	626.825	626.366	622.405
11	11	641.534		641.534	648.968	647.893	643.791	635.315	632.977	632.489	628.962
12	12	621.302		621.302	623.936	623.369	622.923	619.845	619.376	620.774	617.015
13	13	626.839		626.839	631.599	631.638	629.52	624.661	623.582	623.409	618.769
14	14	632.158		632.158	636.615	636.011	633.35	628.009	625.741	625.659	621.019
15	15	624.413		624.413	627.821	626.998	626.201	622.253	621.565	622.504	618.253
16	16	632.464		632.464	638.092	637.61	636.146	631.252	628.861	628.538	623.272
17	17	627.672		627.672	632.106	631.55	630.49	626.444	625.604	627.066	623.183
18	18	626.396		626.396	631.398	633.382	632.287	627.236	624.913	625.357	621.594
19	19	625.316		625.316	629.185	627.788	626.353	622.46	621.152	622.326	618.354
20	20	632.164		632.164	636.976	642.047	637.844	630.441	627.817	628.675	624.176
21	21	634.213		634.213	639.453	639.397	636.221	628.94	626.112	626.627	622.726
22	22	626.318		626.318	630.685	630.368	629.116	624.953	622.92	624.082	621.695
23	23	637.44		637.44	642.611	641.631	639.792	633.928	632.207	632.133	627.399
24	24	640.58		640.58	645.287	641.447	638.119	632.24	629.829	628.914	623.459
25	25	621.76		621.76	625.465	624.509	622.3	618.417	616.711	617.951	614.686
26	26	624.192		624.192	628.793	628.379	626.548	621.177	619.84	621.433	617.83
27	27	635.778		635.778	641.202	639.214	635.808	629.473	628.641	626.403	620.712
28	28	629.223		629.223	634.875	633.139	630.791	625.717	623.529	623.799	619.494
29	29	640.143		640.143	645.692	643.936	639.361	632.148	629.796	629.92	625.843
30	30	627.673		627.673	631.543	631.955	628.101	621.662	620.426	621.716	618.172
31	31	638.059		638.059	643.25	642.342	638.439	629.928	628.69	628.306	625.076
32	32	627.257		627.257	631.753	632.418	631.445	627.09	625.157	626.063	622.164
33	33	612.859		612.859	614.692	613.99	613.485	611.195	611.219	613.5	610.783
34	34	613.291		613.291	615.24	614.582	614.1	611.583	611.688	613.648	611.294
35	35	612.949		612.949	614.682	614.13	613.757	611.209	611.295	613.338	611.149
36	36	635.509									
37	37	626.648									

	L	M	N	O	P	Q	R	S	T	U	V
1	621.546	620.111	620.15	621.517	625.562	630.124	634.294	635.782	635.037	632.439	630.841
2	615.006	614.209	614.275	614.758	616.444	618.719	620.76	620.726	620.19	618.767	618.617
3	621.466	620.772	623.881	631.625	645.33	655.266	656.852	651.074	646.495	641.616	639.526
4	617.136	616.344	616.76	617.485	619.871	622.169	624.195	624.485	624.486	622.92	622.27
5	620.786	619.615	619.689	621.981	626.736	632.582	636.796	637.42	636.96	633.116	632.636
6	615.387	614.526	614.562	615.233	616.724	619.103	621.094	620.806	620.734	619.541	619.723
7	621.721	620.514	621.731	624.685	630.074	634.906	637.592	637.389	636.017	633.552	631.612
8	623.26	622.575	624.337	629.161	636.794	643.781	646.235	644.495	641.131	637.521	634.341
9	616.695	615.577	615.21	615.088	616.271	617.916	619.866	620.988	622.094	621.422	621.397
10	620.837	620.048	621.361	624.702	631.127	635.573	638.111	636.884	635.491	634.315	633.218
11	626.571	625.704	627.644	634.549	644.794	653.681	657.819	654.64	650.026	645.468	644.124
12	616.163	615.385	614.703	614.56	615.508	616.5	618.171	618.491	618.902	618.294	618.705
13	617.375	616.244	615.869	615.664	617.964	620.233	622.959	625.726	626.799	625.186	625.689
14	619.509	618.418	619.074	620.843	625.619	630.814	634.846	635.576	635.82	633.561	632.389
15	617.28	616.309	615.819	615.491	616.437	619.359	619.842	620.089	620.07	619.224	619.733
16	621.546	620.288	619.544	620.036	623.407	627.756	633.077	635.032	635.383	633.544	631.971
17	621.744	620.551	619.595	620.077	622.684	625.391	628.896	630.137	630.564	629.216	628.832
18	620.236	619.418	619.216	620.531	623.837	627.665	631.543	635.122	633.741	632.095	634.532
19	617.136	616.639	616.332	617.125	618.848	620.694	623.204	624.197	624.521	623.822	623.751
20	622.008	620.721	620.349	622.532	627.034	632.265	638.082	637.96	635.388	632.226	631.607
21	621.395	620.608	621.838	624.567	632.235	636.992	640.4	640.87	636.839	633.097	631.088
22	619.971	619.219	620.001	621.427	626.283	630.361	634.625	635.206	634.776	632.733	632.309
23	624.602	622.912	622.226	623.751	628.21	634.427	637.839	638.758	638.5	636.247	634.961
24	621.046	620.175	619.907	622.418	625.555	628.457	630.639	631.26	630.995	629.497	628.699
25	613.638	613.642	613.889	614.968	617.578	619.468	621.179	620.903	620.296	619.092	618.1
26	616.401	615.96	616.819	619.566	624.981	629.446	631.439	630.323	628.133	625.712	624.269
27	618.804	617.808	618.717	620.999	625.685	630.167	634.346	635.223	634.461	632.058	629.582
28	617.65	616.882	617.062	617.662	620.126	623.66	626.916	628.57	628.349	627.145	625.594
29	623.107	622.299	623.627	626.91	632.136	639.077	643.633	642.923	641.273	638.693	636.58
30	616.897	616.045	616.307	617.915	621.616	624.478	627.002	626.073	624.674	622.854	623.56
31	622.326	622.378	623.579	628.546	639.726	645.054	646.713	643.964	639.739	634.93	632.977
32	620.538	619.42	619.22	620.291	623.264	627.419	631.21	632.799	633.046	630.471	628.818
33	610.675	610.208	610.321	609.762	610.023	610.096	611.015	610.789	610.896	610.653	610.883
34	610.951	610.516	610.927	610.294	610.362	610.862	611.871	611.546	611.291	611.166	611.631
35	610.563	610.567	610.675	610.209	610.219	610.453	611.206	610.951	610.826	610.623	611.129

Initial grid output using the provided excel formula.

To work with the data, select the newly arranged dataset and paste it into a new sheet, leaving the top row and first column empty, using 'Paste Special' -> 'Paste Values'. Name this sheet 'Labeled Data'.

Add the labels that correspond to each ROI. At this point, each column represents a time point, and each row represents an ROI.

V38

	A	B	C	D	E	F	G	H	I	J
1		2	4	6	8	10	12	14	16	18
2	CCA1-1	631.216	635.509	635.955	634.256	629.087	626.782	627.304	623.294	621.546
3	CCA1-2	623.317	626.648	625.756	623.651	619.821	618.792	619.927	616.223	615.006
4	CCA1-3	645.794	653.463	651.262	643.889	635.124	630.966	629.383	623.698	621.466
5	CCA1-4	628.615	629.908	628.556	626.056	622.779	621.762	622.802	618.441	617.136
6	CCA1-5	630.76	637.443	638.851	635.565	628.682	626.28	627.029	622.894	620.786
7	CCA1-6	620.988	624.235	623.9	622.372	618.858	618.24	619.76	616.209	615.387
8	CCA1-7	643.175	648.162	644.684	640.36	634.053	631.197	630.158	624.464	621.721
9	CCA1-8	634.566	639.494	638.363	635.134	630.438	629.333	629.852	625.481	623.26
10	CCA1-9	623.806	627.815	627.312	626.237	622.669	621.553	622.404	618.457	616.695
11	CCA1-10	638.265	642.281	639.628	635.267	628.98	626.825	626.366	622.405	620.837
12	CCA1-11	641.534	648.968	647.893	643.791	635.315	632.977	632.489	628.962	626.571
13	CCA1-12	621.302	623.936	623.369	622.923	619.845	619.376	620.774	617.015	616.163
14	CCA1-13	626.839	631.599	631.638	629.52	624.661	623.582	623.409	618.769	617.375
15	CCA1-14	632.158	636.615	636.011	633.35	628.009	625.741	625.659	621.019	619.509
16	CCA1-15	624.413	627.821	626.998	626.201	622.253	621.565	622.504	618.253	617.28
17	CCA1-16	632.464	638.092	637.61	636.146	631.252	628.861	628.538	623.272	621.546
18	CCA1-17	627.672	632.106	631.55	630.49	626.444	625.604	627.066	623.183	621.744
19	CCA1-18	626.396	631.398	633.382	632.287	627.236	624.913	625.357	621.594	620.236
20	CCA1-19	625.316	629.185	627.788	626.353	622.46	621.152	622.326	618.354	617.136
21	CCA1-20	632.164	636.976	642.047	637.844	630.441	627.817	628.675	624.176	622.008
22	CCA1-21	634.213	639.453	639.397	636.221	628.94	626.112	626.627	622.726	621.395
23	CCA1-22	626.318	630.685	630.368	629.116	624.953	622.92	624.082	621.695	619.971
24	CCA1-23	637.44	642.611	641.631	639.792	633.928	632.207	632.133	627.399	624.602
25	CCA1-24	640.58	645.287	641.447	638.119	632.24	629.829	628.914	623.459	621.046
26	CCA1-25	621.76	625.465	624.509	622.3	618.417	616.711	617.951	614.686	613.638
27	CCA1-26	624.192	628.793	628.379	626.548	621.177	619.84	621.433	617.83	616.401
28	CCA1-27	635.778	641.202	639.214	635.808	629.473	628.641	626.403	620.712	618.804
29	CCA1-28	629.223	634.875	633.139	630.791	625.717	623.529	623.799	619.494	617.65
30	CCA1-29	640.143	645.692	643.936	639.361	632.148	629.796	629.92	625.843	623.107
31	CCA1-30	627.673	631.543	631.955	628.101	621.662	620.426	621.716	618.172	616.897
32	CCA1-31	638.059	643.25	642.342	638.439	629.928	628.69	628.306	625.076	622.326
33	CCA1-32	627.257	631.753	632.418	631.445	627.09	625.157	626.063	622.164	620.538
34	Blank-1	612.859	614.692	613.99	613.485	611.195	611.219	613.5	610.783	610.675
35	Blank-2	613.291	615.24	614.582	614.1	611.583	611.688	613.648	611.294	610.951
36	Blank-3	612.949	614.682	614.13	613.757	611.209	611.295	613.338	611.149	610.563
37										
38										

Initial Grid | Labeled Data

Moving the grid to a new sheet and labeling rows and columns.

After entering the grid in a new sheet, separate the rows that correspond to the blanks by moving them down a row. Take the average blank value for the first time point using the AVERAGE command.

30	CCA1-29	640.143	645.692
31	CCA1-30	627.673	631.543
32	CCA1-31	638.059	643.25
33	CCA1-32	627.257	631.753
34			
35	Blank-1	612.859	614.692
36	Blank-2	613.291	615.24
37	Blank-3	612.949	614.682
38	Avg_blanl	=Average(B35:B37)	

Finding the average blank value for the first timepoint.

Drag this across all the columns to get an average blank value for each time point.

28	CCA1-27	635.778	641.202	639.214	635.808	629.473	628.641	626.403	620.712
29	CCA1-28	629.223	634.875	633.139	630.791	625.717	623.529	623.799	619.494
30	CCA1-29	640.143	645.692	643.936	639.361	632.148	629.796	629.92	625.843
31	CCA1-30	627.673	631.543	631.955	628.101	621.662	620.426	621.716	618.172
32	CCA1-31	638.059	643.25	642.342	638.439	629.928	628.69	628.306	625.076
33	CCA1-32	627.257	631.753	632.418	631.445	627.09	625.157	626.063	622.164
34									
35	Blank-1	612.859	614.692	613.99	613.485	611.195	611.219	613.5	610.783
36	Blank-2	613.291	615.24	614.582	614.1	611.583	611.688	613.648	611.294
37	Blank-3	612.949	614.682	614.13	613.757	611.209	611.295	613.338	611.149
38	Average_blank	613.033	614.8713	614.234	613.7807	611.329	611.4007	613.4953	611.0753

Averaging blank values across all time points.

Open a new sheet and name it "Background Corrected Data". Paste in the top row and first column from the 'Labeled Data' sheet. Now, subtract the average blank value from each measurement at every timepoint. The formula for this

using the sample data is
='Labeled Data'!B2-'Labeled Data'!B$38

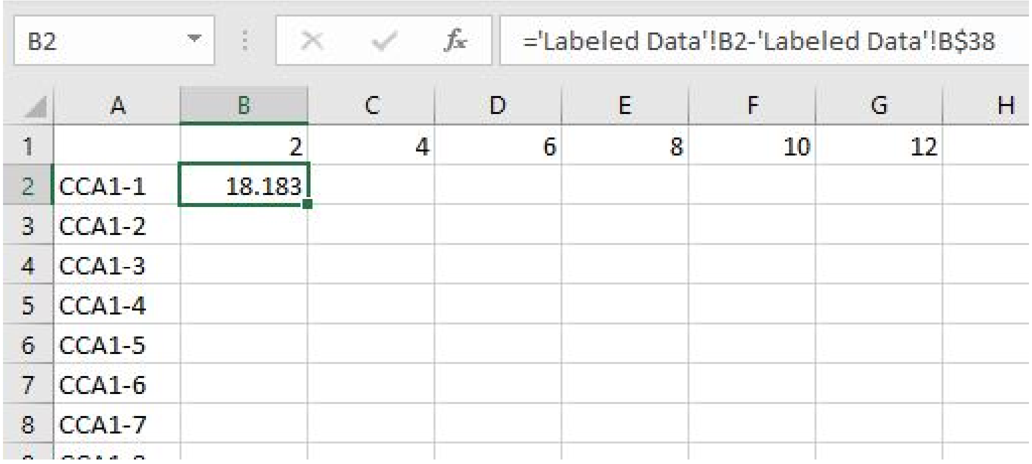

B2 | ='Labeled Data'!B2-'Labeled Data'!B$38

	A	B	C	D	E	F	G	H
1		2	4	6	8	10	12	
2	CCA1-1	18.183						
3	CCA1-2							
4	CCA1-3							
5	CCA1-4							
6	CCA1-5							
7	CCA1-6							
8	CCA1-7							

Formula used to correct for average background for each ROI at each timepoint.

Now, drag it through the whole grid for all ROIs and timepoints (though you can disregard the blank rows). This should output a grid where each ROI mean gray value has the average background values subtracted from it.

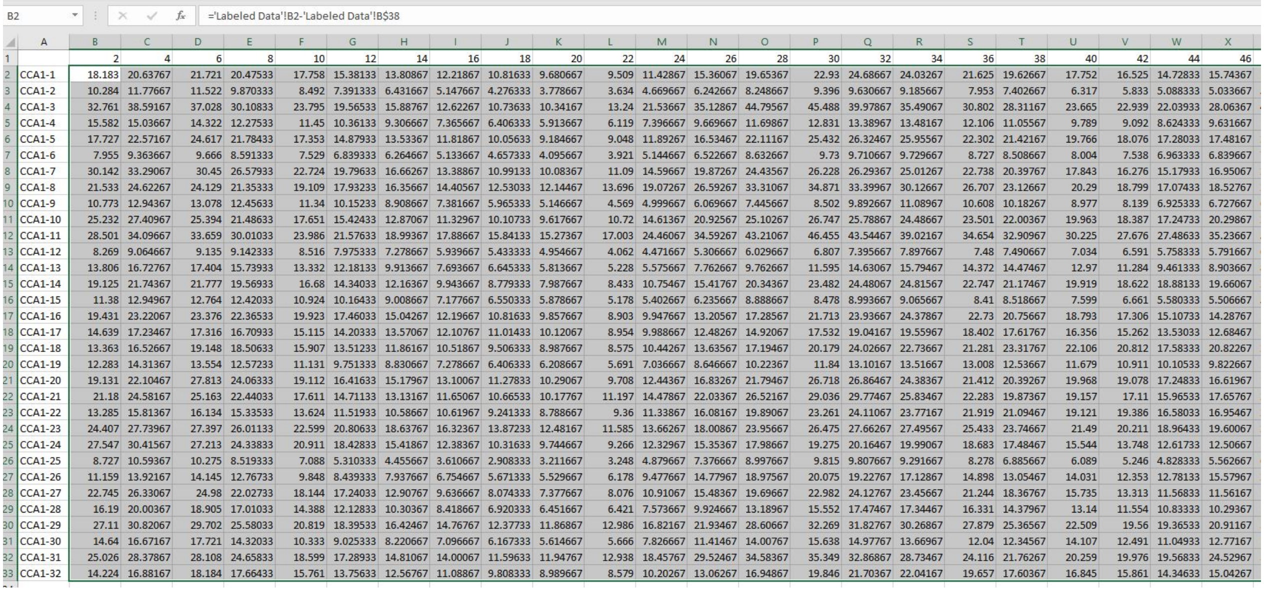

B2 | ='Labeled Data'!B2-'Labeled Data'!B$38

	A	B	C	D	E	F	G	H	I	J	K	L
1		2	4	6	8	10	12	14	16	18	20	22
2	CCA1-1	18.183	20.63767	21.721	20.47533	17.758	15.38133	13.80867	12.21867	10.81633	9.680667	9.509
3	CCA1-2	10.284	11.77667	11.522	9.870333	8.492	7.391333	6.431667	5.147667	4.276333	3.778667	3.634
4	CCA1-3	32.761	38.59167	37.028	30.10833	23.795	19.56533	15.88767	12.62267	10.73633	10.34167	13.24
5	CCA1-4	15.582	15.03667	14.322	12.27533	11.45	10.36133	9.306667	7.365667	6.406333	5.913667	6.119
6	CCA1-5	17.727	22.57167	24.617	21.78433	17.353	14.87933	13.53367	11.81867	10.05633	9.184667	9.048
7	CCA1-6	7.955	9.363667	9.666	8.591333	7.529	6.839333	6.264667	5.133667	4.657333	4.095667	3.921
8	CCA1-7	30.142	33.29067	30.45	26.57933	22.724	19.79633	16.66267	13.38867	10.99133	10.08367	11.09
9	CCA1-8	21.533	24.62267	24.129	21.35333	19.109	17.93233	16.35667	14.40567	12.53033	12.14467	13.696
10	CCA1-9	10.773	12.94367	13.078	12.45633	11.34	10.15233	8.908667	7.381667	5.965333	5.146667	4.569
11	CCA1-10	25.232	27.40967	25.394	21.48633	17.651	15.42433	12.87067	11.32967	10.10733	9.617667	10.72
12	CCA1-11	28.501	34.09667	33.659	30.01033	23.986	21.57633	18.99367	17.88667	15.84133	15.27367	17.003
13	CCA1-12	8.269	9.064667	9.135	9.142333	8.516	7.975333	7.278667	5.939667	5.433333	4.954667	4.062
14	CCA1-13	13.806	16.72767	17.404	15.73933	13.332	12.18133	9.913667	7.693667	6.645333	5.813667	5.228
15	CCA1-14	19.125	21.74367	21.777	19.56933	16.68	14.34033	12.16367	9.943667	8.779333	7.987667	8.433
16	CCA1-15	11.38	12.94967	12.764	12.42033	10.924	10.16433	9.008667	7.177667	6.550333	5.878667	5.178
17	CCA1-16	19.431	23.22067	23.376	22.36533	19.923	17.46033	15.04267	12.19667	10.81633	9.857667	8.903
18	CCA1-17	14.639	17.23467	17.316	16.70933	15.115	14.20333	13.57067	12.10767	11.01433	10.12067	8.954
19	CCA1-18	13.363	16.52667	19.148	18.50633	15.907	13.51233	11.86167	10.51867	9.506333	8.987667	8.575
20	CCA1-19	12.283	14.31367	13.554	12.57233	11.131	9.751333	8.830667	7.278667	6.406333	6.208667	5.691
21	CCA1-20	19.131	22.10467	27.813	24.06333	19.112	16.41633	15.17967	13.10067	11.27833	10.29067	9.708
22	CCA1-21	21.18	24.58167	25.163	22.44033	17.611	14.71133	13.13167	11.65067	10.66533	10.17767	11.197
23	CCA1-22	13.285	15.81367	16.134	15.33533	13.624	11.51933	10.58667	10.61967	9.241333	8.788667	9.36
24	CCA1-23	24.407	27.73967	27.397	26.01133	22.599	20.80633	18.63767	16.32367	13.87233	12.48167	11.585
25	CCA1-24	27.547	30.41567	27.213	24.33833	20.911	18.42833	15.41867	12.38367	10.31633	9.744667	9.266
26	CCA1-25	8.727	10.59367	10.275	8.519333	7.088	5.310333	4.455667	3.610667	2.908333	3.211667	3.248
27	CCA1-26	11.159	13.92167	14.145	12.76733	9.848	8.439333	7.937667	6.754667	5.671333	5.529667	6.178
28	CCA1-27	22.745	26.33067	24.98	22.02733	18.144	17.24033	12.90767	9.636667	8.074333	7.377667	8.076
29	CCA1-28	16.19	20.00367	18.905	17.01033	14.388	12.12833	10.30367	8.418667	6.920333	6.451667	6.421
30	CCA1-29	27.11	30.82067	29.702	25.58033	20.819	18.39533	16.42467	14.76767	12.37733	11.86867	12.986
31	CCA1-30	14.64	16.67167	17.721	14.32033	10.333	9.025333	8.220667	7.096667	6.167333	5.614667	5.666
32	CCA1-31	25.026	28.37867	28.108	24.65833	18.599	17.28933	14.81067	14.00067	11.59633	11.94767	12.938
33	CCA1-32	14.224	16.88167	18.184	17.66433	15.761	13.75633	12.56767	11.08867	9.808333	8.989667	8.579

	A	M	N	O	P	Q	R	S	T	U	V	W	X
1		24	26	28	30	32	34	36	38	40	42	44	46
2	CCA1-1	11.42867	15.36067	19.65367	22.93	24.68667	24.03267	21.625	19.62667	17.752	16.525	14.72833	15.74367
3	CCA1-2	4.669667	6.242667	8.248667	9.396	9.630667	9.185667	7.953	7.402667	6.317	5.833	5.088333	5.033667
4	CCA1-3	21.53667	35.12867	44.79567	45.488	39.97867	35.49067	30.802	28.31167	23.665	22.939	22.03933	28.06367
5	CCA1-4	7.396667	9.669667	11.69867	12.831	13.38967	13.48167	12.106	11.05567	9.789	9.092	8.624333	9.631667
6	CCA1-5	11.89267	16.53467	22.11167	25.432	26.32467	25.95567	22.302	21.42167	19.766	18.076	17.28033	17.48167
7	CCA1-6	5.144667	6.522667	8.632667	9.73	9.710667	9.729667	8.727	8.508667	8.004	7.538	6.963333	6.839667
8	CCA1-7	14.59667	19.87267	24.43567	26.228	26.29367	25.01267	22.738	20.39767	17.843	16.276	15.17933	16.95067
9	CCA1-8	19.07267	26.59267	33.31067	34.871	33.39967	30.12667	26.707	23.12667	20.29	18.799	17.07433	18.52767
10	CCA1-9	4.999667	6.069667	7.445667	8.502	9.892667	11.08967	10.608	10.18267	8.977	8.139	6.925333	6.727667
11	CCA1-10	14.61367	20.92567	25.10267	26.747	25.78867	24.48667	23.501	22.00367	19.963	18.387	17.24733	20.29867
12	CCA1-11	24.46067	34.59267	43.21067	46.455	43.54467	39.02167	34.654	32.90967	30.225	27.676	27.48633	35.23667
13	CCA1-12	4.471667	5.306667	6.029667	6.807	7.395667	7.897667	7.48	7.490667	7.034	6.591	5.758333	5.791667
14	CCA1-13	5.575667	7.762667	9.762667	11.595	14.63067	15.79467	14.372	14.47467	12.97	11.284	9.461333	8.903667
15	CCA1-14	10.75467	15.41767	20.34367	23.482	24.48067	24.81567	22.747	21.17467	19.919	18.622	18.88133	19.66067
16	CCA1-15	5.402667	6.235667	8.888667	8.478	8.993667	9.065667	8.41	8.518667	7.599	6.661	5.580333	5.506667
17	CCA1-16	9.947667	13.20567	17.28567	21.713	23.93667	24.37867	22.73	20.75667	18.793	17.306	15.10733	14.28767
18	CCA1-17	9.988667	12.48267	14.92067	17.532	19.04167	19.55967	18.402	17.61767	16.356	15.262	13.53033	12.68467
19	CCA1-18	10.44267	13.63567	17.19467	20.179	24.02667	22.73667	21.281	23.31767	22.106	20.812	17.58333	20.82267
20	CCA1-19	7.036667	8.646667	10.22367	11.84	13.10167	13.51667	13.008	12.53667	11.679	10.911	10.10533	9.822667
21	CCA1-20	12.44367	16.83267	21.79467	26.718	26.86467	24.38367	21.412	20.39267	19.968	19.078	17.24833	16.61967
22	CCA1-21	14.47867	22.03367	26.52167	29.036	29.77467	25.83467	22.283	19.87367	19.157	17.11	15.96533	17.65767
23	CCA1-22	11.33867	16.08167	19.89067	23.261	24.11067	23.77167	21.919	21.09467	19.121	19.386	16.58033	16.95467
24	CCA1-23	13.66267	18.00867	23.95667	26.475	27.66267	27.49567	25.433	23.74667	21.49	20.211	18.96433	19.60067
25	CCA1-24	12.32967	15.35367	17.98667	19.275	20.16467	19.99067	18.683	17.48467	15.544	13.748	12.61733	12.50667
26	CCA1-25	4.879667	7.376667	8.997667	9.815	9.807667	9.291667	8.278	6.885667	6.089	5.246	4.828333	5.562667
27	CCA1-26	9.477667	14.77967	18.97567	20.075	19.22767	17.12867	14.898	13.05467	14.031	12.353	12.78133	15.57967
28	CCA1-27	10.91067	15.48367	19.69667	22.982	24.12767	23.45667	21.244	18.36767	15.735	13.313	11.56833	11.56167
29	CCA1-28	7.573667	9.924667	13.18967	15.552	17.47467	17.34467	16.331	14.37967	13.14	11.554	10.83333	10.29367
30	CCA1-29	16.82167	21.93467	28.60667	32.269	31.82767	30.26867	27.879	25.36567	22.509	19.56	19.36533	20.91167
31	CCA1-30	7.826667	11.41467	14.00767	15.638	14.97767	13.66967	12.04	12.34567	14.107	12.491	11.04933	12.77167
32	CCA1-31	18.45767	29.52467	34.58367	35.349	32.86867	28.73467	24.116	21.76267	20.259	19.976	19.56833	24.52967
33	CCA1-32	10.20267	13.06267	16.94867	19.846	21.70367	22.04167	19.657	17.60367	16.845	15.861	14.34633	15.04267

Calculating background corrected values for the entire dataset.

Paste this grid into a new sheet called BCD Values using 'Paste Special' --> 'Values Only'. Then, make another new sheet labeled 'For Biodare'. Paste the grid from the BCD Values sheet into this grid using the 'Transpose' option.

	A	B	C	D	E	F	G	H	I	J	K
1		CCA1-1	CCA1-2	CCA1-3	CCA1-4	CCA1-5	CCA1-6	CCA1-7	CCA1-8	CCA1-9	CCA1
2	2	18.183	10.284	32.761	15.582	17.727	7.955	30.142	21.533	10.773	25
3	4	20.63767	11.77667	38.59167	15.03667	22.57167	9.363667	33.29067	24.62267	12.94367	27.4
4	6	21.721	11.522	37.028	14.322	24.617	9.666	30.45	24.129	13.078	25
5	8	20.47533	9.870333	30.10833	12.27533	21.78433	8.591333	26.57933	21.35333	12.45633	21.4
6	10	17.758	8.492	23.795	11.45	17.353	7.529	22.724	19.109	11.34	17
7	12	15.38133	7.391333	19.56533	10.36133	14.87933	6.839333	19.79633	17.93233	10.15233	15.4
8	14	13.80867	6.431667	15.88767	9.306667	13.53367	6.264667	16.66267	16.35667	8.908667	12.8
9	16	12.21867	5.147667	12.62267	7.365667	11.81867	5.133667	13.38867	14.40567	7.381667	11.3
10	18	10.81633	4.276333	10.73633	6.406333	10.05633	4.657333	10.99133	12.53033	5.965333	10.1
11	20	9.680667	3.778667	10.34167	5.913667	9.184667	4.095667	10.08367	12.14467	5.146667	9.61
12	22	9.509	3.634	13.24	6.119	9.048	3.921	11.09	13.696	4.569	1
13	24	11.42867	4.669667	21.53667	7.396667	11.89267	5.144667	14.59667	19.07267	4.999667	14.6
14	26	15.36067	6.242667	35.12867	9.669667	16.53467	6.522667	19.87267	26.59267	6.069667	20.9
15	28	19.65367	8.248667	44.79567	11.69867	22.11167	8.632667	24.43567	33.31067	7.445667	25.1
16	30	22.93	9.396	45.488	12.831	25.432	9.73	26.228	34.871	8.502	26
17	32	24.68667	9.630667	39.97867	13.38967	26.32467	9.710667	26.29367	33.39967	9.892667	25.7
18	34	24.03267	9.185667	35.49067	13.48167	25.95567	9.729667	25.01267	30.12667	11.08967	24.4
19	36	21.625	7.953	30.802	12.106	22.302	8.727	22.738	26.707	10.608	23
20	38	19.62667	7.402667	28.31167	11.05567	21.42167	8.508667	20.39767	23.12667	10.18267	22.0
21	40	17.752	6.317	23.665	9.789	19.766	8.004	17.843	20.29	8.977	19
22	42	16.525	5.833	22.939	9.092	18.076	7.538	16.276	18.799	8.139	18
23	44	14.72833	5.088333	22.03933	8.624333	17.28033	6.963333	15.17933	17.07433	6.925333	17.2
24	46	15.74367	5.033667	28.06367	9.631667	17.48167	6.839667	16.95067	18.52767	6.727667	20.2
25	48	18.70133	5.851333	46.34533	12.00533	21.65133	7.731333	22.18833	23.75733	6.768333	28.5
26	50	24.74867	7.480667	68.84467	15.38967	30.38667	9.816667	30.23567	32.59767	7.200667	37.8
27	52	32.796	9.285	80.852	17.226	36.454	11.95	38.507	40.109	8.243	47
28	54	38.831	11.363	81.579	19.663	40.924	13.394	45.296	43.244	10.757	51
29	56	40.25067	12.80267	74.09367	20.59267	41.26267	14.17167	47.47367	42.90967	12.66067	50.6
30	58	38.25933	13.69633	62.79033	20.51433	38.70233	14.36533	46.38433	39.77333	14.40333	46.0
31	60	34.9	13.381	50.9	19.567	36.058	13.851	43.123	35.668	15.066	42
32	62	31.71767	12.89067	44.29267	18.65267	34.31667	13.74267	40.49367	32.40667	14.64967	37.6
33	64	27.971	12.072	38.927	17.737	32.801	13.228	37.21	29.49	13.966	33
34	66	24.674	11.496	34.448	16.819	28.603	12.993	34.441	25.002	12.985	3
35	68	22.52967	11.29867	34.24967	16.47367	26.38367	12.34167	32.43467	21.97267	11.97867	29.9
36	70	23.76167	10.87167	44.77167	17.95267	28.68967	12.20967	32.82867	21.71667	11.31667	38.1
37	72	32.621	12.555	67.12	23.804	34.483	13.974	38.152	26.011	11.403	53
38	74	46.23233	14.68333	90.34733	28.93933	43.53933	17.23833	47.11533	32.17933	12.24933	74.8

Initial Grid | Labeled Data | Background Corrrected Data | BCD (values) | **For Biodare**

Transposed values for analysis in BioDare.

Prior to uploading it to Biodare, paste the 'For Biodare' sheet in to a new excel file.

For Biodare to conduct analyses, all ROIs from the same genotype/treatment must have the same name, with no unique identifier. As a final step, ensure that all columns from a single genotype or treatment group have the same name. In the sample dataset, all plants are *CCA1p:LUC*, so all get the column name CCA1. (as an example, if we also had five wild type plants, we would name those five columns WT).

	CCA1	CCA1	CCA1	CCA1	CCA1	CCA1	CCA1	CCA1	CCA1	CCA1	CCA1	CCA1	CCA1	CCA1	CCA1	CCA1	CCA1	CCA1
2	18.183	10.284	32.761	15.582	17.727	7.955	30.142	21.533	10.773	25.232	28.501	8.269	13.806	19.125	11.38	19.431	14.639	13.363
4	20.63767	11.77667	38.59167	15.03667	22.57167	9.363667	33.29067	24.62267	12.94367	27.40967	34.09667	9.064667	16.72767	21.74367	12.94967	23.22067	17.23467	16.52667
6	21.721	11.522	37.028	14.322	24.617	9.666	30.45	24.129	13.078	25.394	33.659	9.135	17.404	21.777	12.764	23.376	17.316	19.148
8	20.47533	9.870333	30.10833	12.27533	21.78433	8.591333	26.57933	21.35333	12.45633	21.48633	30.01033	9.142333	15.73933	19.56933	12.42033	22.36533	16.70933	18.50633
10	17.758	8.492	23.795	11.45	17.353	7.529	22.724	19.109	11.34	17.651	23.986	8.516	13.332	16.68	10.924	19.923	15.115	15.907
12	15.38133	7.391333	19.56533	10.36133	14.87933	6.839333	19.79633	17.93233	10.15233	15.42433	21.57633	7.975333	12.18133	14.34033	10.16433	17.46033	14.20333	13.51233
14	13.80867	6.431667	15.88767	9.306667	13.53367	6.264667	16.66267	16.35667	8.908667	12.87067	18.99367	7.278667	9.913667	12.16367	9.008667	15.04267	13.57067	11.86167
16	12.21867	5.147667	12.62267	7.365667	11.81867	5.133667	13.38867	14.40567	7.381667	11.32967	17.88667	5.939667	7.693667	9.943667	7.177667	12.19667	12.10767	10.51867
18	10.81633	4.276333	10.73633	6.406333	10.05633	4.657333	10.99133	12.53033	5.965333	10.10733	15.84133	5.433333	6.645333	8.779333	6.550333	10.81633	11.01433	9.506333
20	9.680667	3.778667	10.34167	5.913667	9.184667	4.095667	10.08367	12.14467	5.146667	9.617667	15.27367	4.954667	5.813667	7.987667	5.878667	9.857667	10.12067	8.987667

Changed column names for analysis.

Data Transformation in R:

Copy the two columns of data from Fiji (the measurement serial number and the measurement (i.e. mean gray value)). Paste these into an Excel Spreadsheet and save as a .csv file.

Download the R script 'FormattingFijiDataOutput.R' from github: **https://github.com/GreenhamLab/CCD_Imaging**

We have also provided the images, ROIs, and Raw Data output from Fiji as a test sample.

Run the script on the Fiji output data to format it for BioDare.

Note: At this time, Biodare adds quotation marks (" ") around the sample names when using a file output by R. This can be fixed by opening the output in excel and saving it as a csv. If we find a solution for this, we will update the script on our github page.

Powered by eLabNext
