# Supplementary material for: Assembly and operation of an imaging system for long-term monitoring of bioluminescent and fluorescent reporters in plants
Source: Plant Methods. 2023 Mar 1;19:19. doi: 10.1186/s13007-023-00997-0 (PMC9976486; doi:10.1186/s13007-023-00997-0)
Supplement: Supplementary file 3 — Additional file 3. Supplemental Figures 1–3. [file 13007_2023_997_MOESM3_ESM.docx]

**Figure S1**

**Supplemental Figure 1: Datalogger temperature logs for Growfilm and Heliospectra imaging**. (a) Temperature logs taken at plate level under the Growfilm white LED lights. (b) Temperature logs taken at plate level under the Heliospectra LED lights.

**Figure S2**

**
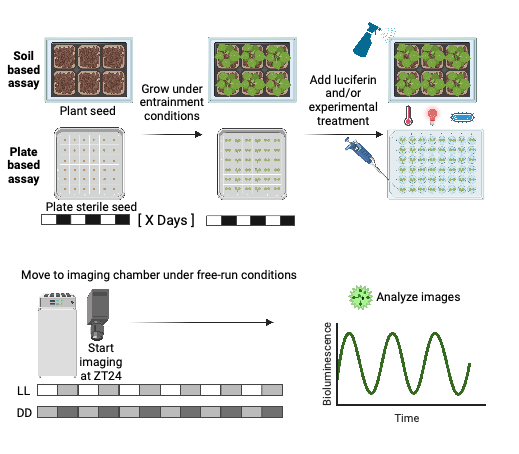
**

**Supplemental Figure 2: Overall schematic of imaging methodology.** Seeds are planted into soil or onto tissue culture plates using sterile technique. Seeds are germinated and grown under entrainment conditions (cycling light and/or temperature) until desired age for experimental treatment. Plants are then sprayed with luciferin for bioluminescent essays as necessary, treated with a biological agent or exposed to a new environmental condition per the experimental design. Seedlings on plates or in pots are then imaged in the chamber to capture bioluminescence or fluorescence as part of a time course. The chamber is set to the desired lighting and temperature conditions, frequently constant lighting and temperature for circadian experiments. Once imaging is complete, the pictures are then analyzed for changes in bioluminescence using downstream image analysis software, such as NIH Image J or Metamorph.

**Figure S3**


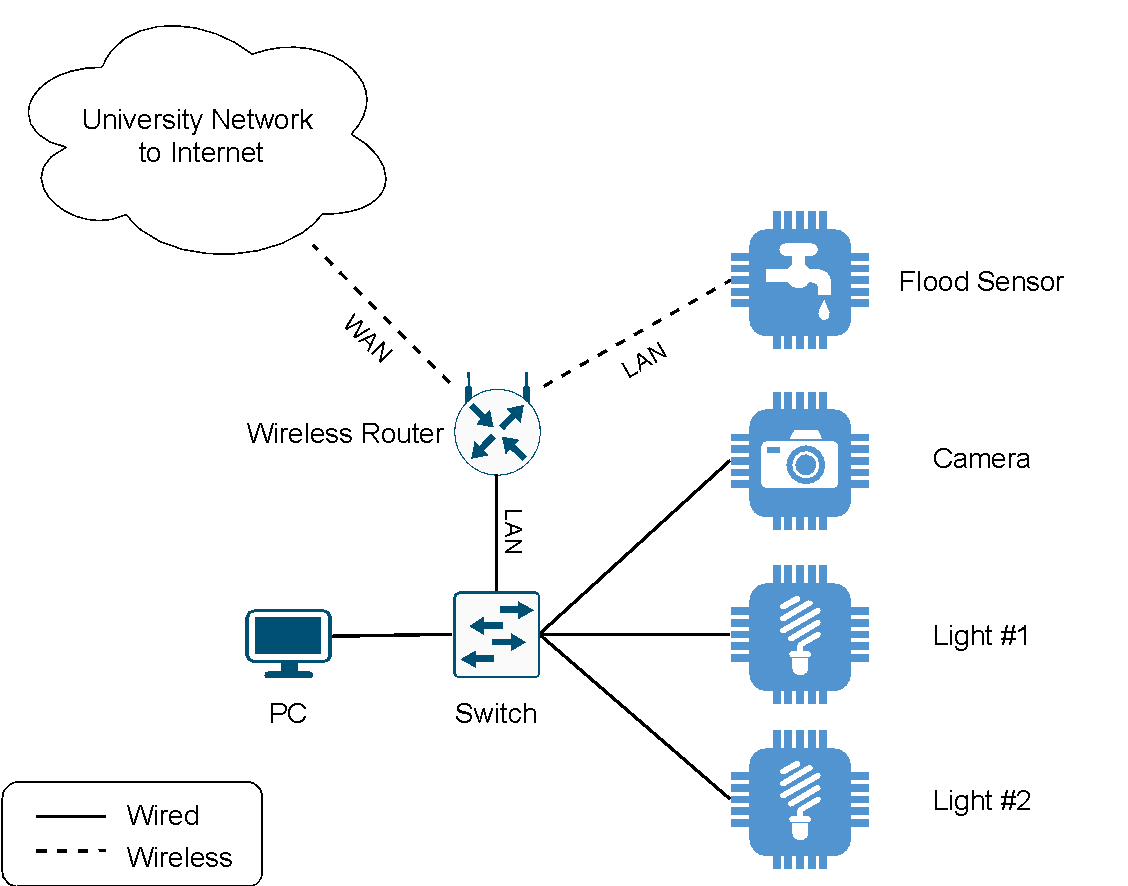


**Supplemental Figure 3:** Schematic of System 2 network setup
